# Targeting the CCL5/CCR5 axis in tumor-stromal crosstalk to overcome cisplatin resistance in neuroendocrine prostate cancer

**DOI:** 10.1186/s13046-025-03552-y

**Published:** 2025-10-28

**Authors:** Bo Liu, Weiwei Zhang, Yiyi Ji, Jiajin Wu, Ruopeng Su, Xinyu Liu, Ang Li, Kai Shen, Xinyu Chai, Haotian Wu, Zehua Ma, Cong Hu, Zhou Jiang, Liang Dong, Yinjie Zhu, Baijun Dong, Wei Xue, Jiahua Pan, Qi Wang

**Affiliations:** 1 Department of Urology, Ren Ji Hospital, Shanghai Jiao Tong University School of Medicine, Shanghai, 200120 China; 2 Department of Pathology, Ren Ji Hospital, Shanghai Jiao Tong University School of Medicine, Shanghai, 200120 China; 3 Shanghai Key Laboratory for Tumor Microenvironment and Inflammation, Shanghai Jiao Tong University School of Medicine, Shanghai, 200120 China; 4 Department of Urology, Guizhou Provincial People’s Hospital, Guiyang, 550001 China

**Keywords:** Neuroendocrine prostate cancer, Cancer-associated fibroblasts, Cisplatin resistance, CCL5, Maraviroc

## Abstract

**Background:**

Neuroendocrine prostate cancer (NEPC) is an aggressive subtype of prostate cancer with limited therapeutic options. Although cisplatin is recommended as a first-line treatment, its clinical efficacy is hindered by the rapid development of drug resistance, highlighting the urgent need for effective strategies to overcome cisplatin resistance.

**Methods:**

We established a NEPC mouse allograft model and performed RNA sequencing to identify genes associated with cisplatin resistance. The role of CCL5 in tumor-stromal crosstalk was investigated using immunofluorescence, ELISA assays, co-culture assays, and CCL5 knockout mice. Mechanistic studies were conducted to explore CCL5/CCR5-mediated signaling pathways. The therapeutic efficacy of cisplatin combined with maraviroc, an FDA-approved CCR5 antagonist, was evaluated in vitro using NEPC cell lines and patient-derived organoids, and in vivo using NEPC mouse models.

**Results:**

Here, we identify a tumor-stromal interaction mediated by the CCL5/CCR5 axis that drives cisplatin resistance in NEPC. Cisplatin-induced DNA damage promotes a cGAS-STING–dependent senescence program in cancer-associated fibroblasts (CAFs), resulting in the secretion of CCL5, a key senescence-associated secretory phenotype factor. CCL5 from CAFs binds to CCR5 on tumor cells, promoting the formation of a CCR5/β-arrestin1/p85 complex that activates the PI3K/AKT pathway. This activation enhances DNA repair, protecting tumor cells from cisplatin-induced apoptosis. Pharmacologic inhibition of the CCL5/CCR5 pathway using maraviroc, an FDA-approved CCR5 antagonist, sensitizes NEPC cells to cisplatin treatment and significantly prolongs survival in NEPC mouse models.

**Conclusions:**

Our findings identify the CCL5/CCR5 axis as a key mediator of tumor-stromal crosstalk driving cisplatin resistance in NEPC. Mechanistically, CAF-derived CCL5 activates AKT signaling in tumor cells by promoting the formation of the CCR5/β-arrestin1/p85 complex. Targeting this pathway with maraviroc in combination with cisplatin offers a promising therapeutic strategy for overcoming drug resistance in NEPC.

**Graphical Abstract:**

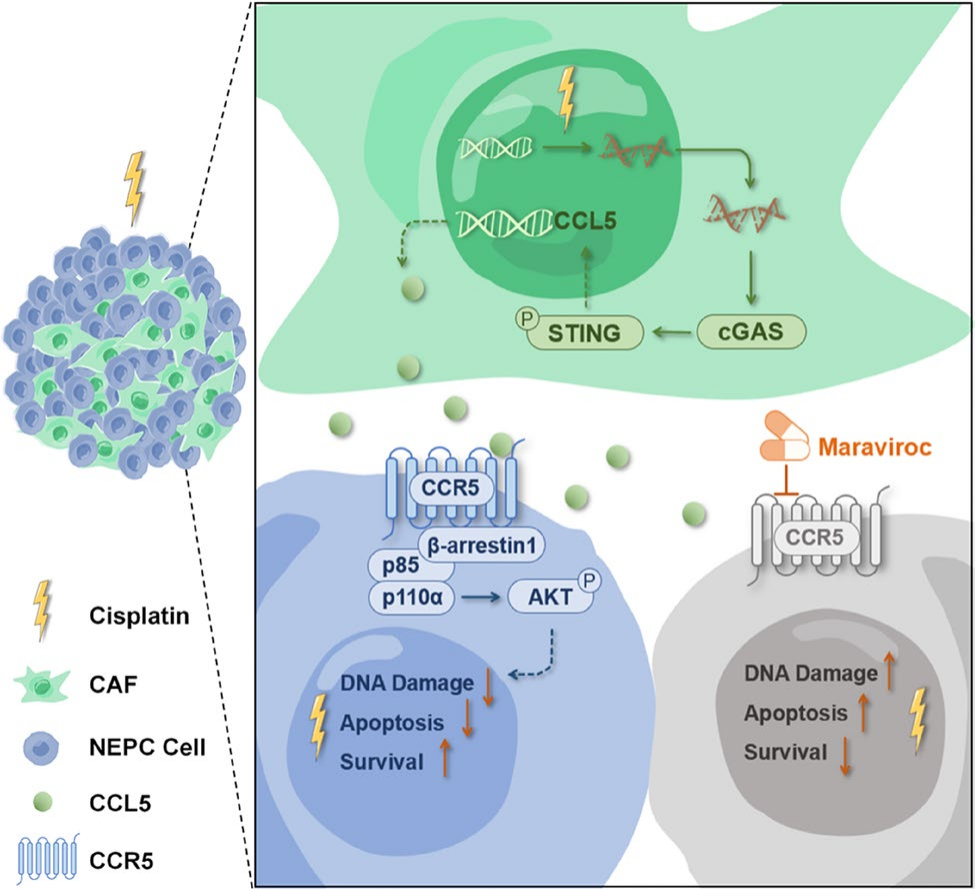

**Supplementary Information:**

The online version contains supplementary material available at 10.1186/s13046-025-03552-y.

## Background

Cisplatin-based chemotherapy is a cornerstone treatment for aggressive and metastatic cancers, yet its effectiveness is frequently undermined by drug resistance [1]. Cisplatin works by forming DNA adducts that trigger the DNA damage response and apoptosis. However, tumors develop resistance through mechanisms such as reduced drug accumulation, enhanced DNA repair, and impaired cell death [2]. Beyond these intrinsic mechanisms, the tumor microenvironment plays an extrinsic role in driving resistance, prompting recent research to focus on intercellular communication within tumor tissues [3, 4].

Cancer-associated fibroblasts (CAFs) are among the most abundant stromal cell populations in the tumor microenvironment, exerting diverse actions in the disease progression [4, 5]. For example, CAFs enhance chemotherapy resistance by secreting IL6 in pancreatic cancer [6]. Conversely, a recent study found that primary CAFs with weaker cancer-promoting properties failed to induce cisplatin resistance in oral squamous cell carcinoma [7]. These findings reflect the complex and context-dependent interactions between CAFs and tumor cells, mediating largely by soluble factors such as chemokines, a family of small, multifunctional chemotactic cytokines best known for recruiting and positioning immune cells from the bloodstream into precise sites [8]. CAFs and tumor cells exploit this system, weaving a complicated network that modulates therapy responses. Understanding the specific components within tumor tissues that regulate these pathways in distinct contexts is essential for improving cisplatin efficacy.

The role of CAF-derived chemokines in therapy resistance is particularly relevant in neuroendocrine prostate cancer (NEPC), an aggressive subtype characterized by histologically-defined small cell morphology and elevated neuroendocrine biomarkers such as synaptophysin, chromogranin A, and CD56 [9, 10]. Recent studies, including ours, have uncovered key pathways in prostate cancer cells that drive neuroendocrine differentiation and resistance to androgen deprivation therapy [11–13]. However, actionable therapeutic strategies for NEPC remain elusive. NEPC patients, who often fail conventional hormone therapy or docetaxel treatment, are recommended cisplatin-based chemotherapy per National Comprehensive Cancer Network guidelines [14]. Unfortunately, cisplatin shows limited efficacy, with patients frequently experiencing rapid drug resistance and tumor relapse. Efforts to investigate how tumor cells and the microenvironment evade cisplatin-induced cell death are hindered partly due to the limited availability of patient-derived tumor samples.

To investigate cisplatin resistance in NEPC, we compared tumor tissues from NEPC mouse models treated with cisplatin or PBS. Our analysis revealed that cisplatin-treated samples preferentially expressed the chemokine CCL5, mediated by the cGAS-STING pathway in CAFs. CCL5 then binds to CCR5 on tumor cells, activating the PI3K/AKT cascade and protecting these cells from cisplatin-induced cell death. Notably, the CCR5 inhibitor maraviroc enhanced the efficacy of cisplatin treatment. Our findings highlight the CCL5/CCR5/AKT signaling as a key driver of cisplatin resistance in NEPC and suggest that targeting it with maraviroc may enhance chemotherapy efficacy.

## Methods

### Cell culture

LASCPC-01 was purchased from the American Type Culture Collection (ATCC) and maintained in HITES: RPMI-1640 (Gibco, USA) containing 10 nM hydrocortisone (Sigma, USA), 5 µg/mL insulin (MedChemExpress, China), 10 µg/mL transferrin (Sigma, USA), 4 ng/mL β-estradiol (Sigma, USA), 3 ng/mL sodium selenite (Sigma, USA), 5% fetal bovine serum (Gibco, USA), and 1% penicillin-streptomycin (Biosharp, China). HEK293T was purchased from the Cell Bank, Shanghai Institutes for Biological Sciences, Chinese Academy of Sciences, cultured in DMEM (Gibco, USA) containing 10% fetal bovine serum (Gibco, USA) and 1% penicillin-streptomycin (Biosharp, China). All cells were grown at 37 °C with 5% CO_2_ and were routinely checked for mycoplasma contamination. Cell line identity was verified using high-resolution short tandem repeat profiling.

### Isolation and culture of primary mouse NEPC cells and cancer-associated fibroblasts

Tumor tissues were enzymatically dissociated into single-cell suspensions and subjected to magnetic-activated cell sorting (MACS)-based separation, as previously described [15, 16]. In brief, tumor tissues from transgenic mice (Pb-Cre4: *Pten*^f/f^; *Trp53*^f/f^; *Rb1*^f/f^) were harvested and immediately placed on ice. After washing with cold PBS, tissues were minced into small pieces (2 mm^3^) and digested in RPMI-1640 containing 0.2 mg/mL collagenase I (17100017, Gibco, USA), 0.2 mg/mL collagenase IV (17104019, Gibco, USA), 0.01 mg/mL DNase I (07900, STEMCELL Technologies, Canada), and 0.1 mg/mL dispase (A002100-0050, Sangon, China) for 2 h at 37 °C with gentle agitation. Cell suspensions were filtered through 70 μm strainers and centrifuged at 300 g for 5 min. Red blood cells were removed by lysis buffer (BD Biosciences, USA) for 5 min, quenched with PBS, and centrifuged again.

For magnetic separation, cells were resuspended in MACS buffer (PBS containing 0.5% bovine serum albumin and 2 mM EDTA) at 300 µL per 10^7^ cells. FcR (CD16/32) blocking reagent (14–0161-82, ebioscience, USA) was added following the manufacturer’s instructions to minimize non-specific binding. EpCAM positive tumor cells were enriched using a two-step labeling procedure: incubation with biotinylated anti-EpCAM antibody (13–9326-82, ebioscience, USA) at 1 µL per 3 × 10^6^ total cells for 1 h at 4 °C, followed by anti-Biotin MicroBeads (130-090-485, Miltenyi Biotec, Germany) at 10 µL per 3 × 10^6^ total cells for 15 min at 4 °C. After labeling, suspensions were processed through MACS LS columns (Miltenyi Biotec, Germany). The flow-through was collected as CAFs, whereas retained EpCAM positive cells (mouse NEPC cells) were eluted after column removal. All labeling and column steps were performed on ice. Human CAFs were isolated from fresh biopsy tissues of two NEPC patients using a similar workflow. The clinical characteristics of these patients are summarized in Table S1.

Isolated mouse NEPC cells and CAFs were maintained in RPMI-1640 with 10% FBS and 1% penicillin-streptomycin (Biosharp, China). NEPC identity was confirmed by immunofluorescence staining for SYP and EpCAM, while CAFs were validated by αSMA expression. CAFs between passages 2–10 were used for experiments.

### Western blot and co-immunoprecipitation

Cells were treated with cisplatin, maraviroc, CCL5, or the indicated agents for 48 h and then harvested for Western blot analysis. Cells or tissues were harvested and lysed in 1% SDS lysis buffer containing a protease and phosphatase inhibitor cocktail. The cell lysates were then analyzed by immunoblotting using the appropriate antibodies. For immunoprecipitation assays, cells were harvested and lysed in a buffer containing 20 mM Tris-HCl, 150 mM NaCl, 1 mM EDTA, and 1% NP-40. The lysates were pre-cleaned and then immunoprecipitated with specific antibodies or IgG at 4 °C for 12 h (antibodies are listed in the Table S2). The protein A/G magnetic beads (HY-K0202, MedChemExpress, China) were added and incubated at 4 °C for 2 h. The immunoprecipitants were washed four times with lysis buffer and subsequently analyzed by immunoblotting. The protein levels were normalized to the GAPDH using ImageJ. Western blot and co-immunoprecipitation experiments were performed using samples collected from independent cell or tissue preparations, and each experiment was repeated three times independently with similar results.

### Quantitative PCR

Total RNA was extracted from cells and tumor tissues using TRIzol Reagent. Reverse transcription was performed using a Reverse Transcription Kit (R201, Vazyme, China). QPCR was conducted with SYBR Green (Q711, Vazyme, China) on a LightCycler 480 qPCR machine (Roche, Switzerland). Relative mRNA expression levels were calculated using the 2^−ΔΔCq^ method. QPCR experiments were performed using cDNA prepared from independent biological samples. Each biological replicate was analyzed in triplicate technical repeats, and the mean of the technical replicates was used for statistical analysis. Primer sequences are provided in Table S3.

### Immunohistochemistry

Tumor tissues were initially fixed in 10% formalin for 48 h, then embedded in paraffin and sectioned into 4 μm slices. These sections were deparaffinized and rehydrated through a graded series of ethanol solutions. Antigen retrieval was conducted using EDTA buffer at 95 °C for 30 min. To inhibit native peroxidase activity, the sections were treated with 3% hydrogen peroxide for 10 min. Non-specific binding was minimized by incubating the sections with 10% horse serum in TBST for 1 h at room temperature. Following this, the tissue sections were incubated overnight at 4 °C with specific primary antibodies diluted in the blocking buffer. The list of antibodies utilized is provided in Table S2. After thorough washing with TBST, the sections were incubated with appropriate secondary antibodies for 1 h at room temperature. Subsequently, the slides were subjected to diaminobenzidine staining for color development. The slides were then counterstained with hematoxylin, dehydrated, and mounted under coverslips. Representative images were obtained using an Olympus microscope. The immunohistochemistry quantitation was performed manually by two independent investigators who were blinded to the experimental groups. All samples within the same batch were processed and scored under identical experimental and staining conditions to ensure consistency. The H-score was determined by multiplying each staining intensity (0 = none, 1 = weak, 2 = moderate, 3 = strong) by the proportion of cells (0–100) displaying that intensity [17, 18]. Scores ranged from 0 to 300, with 300 representing all cells exhibiting strong (3+) staining. Immunohistochemistry staining was analyzed across multiple fields of view as technical replicates and independently repeated three times, yielding consistent results.

### Immunofluorescence

For immunofluorescence staining, cells were seeded onto glass coverslips in 12-well plates and treated with cisplatin, CCL5, maraviroc, or indicated agents for 48 h.The cells were fixed with 4% paraformaldehyde in PBS for 30 min at room temperature. Permeabilization was performed using 0.15% Triton X-100 in PBS for 10 min. To block non-specific binding, cells were incubated with 5% bovine serum albumin in PBS for 30 min at room temperature. Primary antibodies were incubated in the cells overnight at 4 °C. Following incubation, cells were washed three times with PBS and then incubated with appropriate fluorophore-conjugated secondary antibodies for 1 h at room temperature in the dark. After three final washes with PBS, the coverslips were mounted onto glass slides using DAPI to counterstain the nuclei. The stained cells were visualized and imaged using a microscope.

For paraffin-embedded tissue staining, antigen retrieval was performed with EDTA solution at 95 °C for 30 min. Tumor sections were blocked by 10% horse serum with 0.3 M glycine for 1 h at room temperature then stained with primary antibodies targeting CCL5 at 4 °C overnight. After thorough washing with TBST, the sections were incubated with secondary antibodies for 1 h at room temperature. Fluorescence-labeled probe (TYR-Cy3, Recordbio, China) was incubated at room temperature for 10 min. Then, antigen retrieval and blocking were repeated once more. The sections were incubated with primary antibodies targeting EpCAM, CD45, or αSMA at 4 °C overnight. After thorough washing with TBST, the sections were incubated with appropriate secondary antibodies for 1 h. Fluorescence-labeled probe (TYR-488, Recordbio, China) was incubated again. Finally, 50 µg/mL DAPI was used for nuclear staining. Colocalization was evaluated using Pearson’s R-value with the Coloc2 plugin in Fiji [19]. Immunofluorescence staining was analyzed across multiple fields of view as technical replicates and independently repeated on tissues from at least three mice, yielding consistent results.

### Senescence-associated β-gal staining

Mouse NEPC cells, mouse CAFs, and human CAFs were seeded in 12-well plates. After the indicated treatments, the cells were washed with PBS and fixed in 4% paraformaldehyde solution for 30 min at room temperature. Then the cells were washed with PBS and incubated with 0.5 mL β-gal staining solution (C0602, Beyotime, China) at 37 °C for 12–24 h. After incubation, cells were washed with PBS and observed under microscope. Senescence-associated β-gal staining was quantified from five technical replicates and independently repeated at least three times with consistent outcomes.

### Flow cytometry

For cell cycle analysis, CAFs were treated with cisplatin, CCL5, maraviroc, or indicated agents for 48 h. The cells were harvested and washed twice with cold PBS. The cell pellet was then fixed in cold 70% ethanol solutions, added dropwise while vortex, and stored at 4 °C overnight. The cells were washed twice with PBS to remove the ethanol and resuspended in 1 mL of PBS containing 50 µg/mL DAPI and incubated at room temperature in the dark for 20 min. Following incubation, the stained cells were filtered through a 40 μm cell strainer to remove clumps and transferred to flow cytometry tubes. The samples were analyzed using a flow cytometer, with DAPI fluorescence detected in the appropriate channel. Cell cycle distribution was determined by analyzing the DNA content using FlowJo (V10.8). For apoptosis detection, mouse NEPC cells were assessed using the Annexin V-FITC/PI Apoptosis Detection Kit (C1062, Beyotime, China). Flow cytometry analyses were performed using FlowJo (V10.8). Flow cytometry analyses were performed on cells from independent biological samples, with at least three biological replicates included per experiment.

### Detection of DNA in cytosolic extracts

The method for cytosolic DNA extraction has been described in detail in our previous publication [20]. Briefly, cytosolic DNA was extracted from gently lysed cells, followed by quantification using qPCR. The gDNA and mtDNA primers used for this analysis are listed in the Table S3.

### ELISA assays

The concentrations of CCL5 in cell supernatants from mouse CAFs and human CAFs were detected using ELISA kits (EMC106 and EHC143, NeoBioscience Technology, China) according to the manufacturer’s instructions. ELISA assays were carried out on samples from independent biological replicates, with each sample measured in triplicate technical replicates.

### Comet assays

Mouse NEPC cells were treated with cisplatin, CCL5, maraviroc, or indicted agents for 48 h and subsequently analyzed using a comet assay kit (4250-050-K, R&D SYSTEMS, USA) following the manufacturer’s instructions. Comet assays were performed with at least three independent biological replicates.

### Conditioned medium assays

Day 0: 5 × 10^5^ mouse CAFs or human CAFs were plated in 6-well plates. Day 1: CAFs were treated with 0.5 µg/mL cisplatin (15663-27-1, Sigma, USA) or PBS for 24 h. Day 2: The medium in each well was replaced with fresh medium for 24 h. Day 3: The conditioned medium was collected and filtered with a 0.45 μm filter to remove cell debris. The supernatants from cisplatin-treated (cisplatin-stimulated conditioned medium) or untreated CAFs (conditioned medium) were mixed with a complete culture medium at a 1:1 ratio. These mixtures were then used to culture tumor cells (mouse NEPC cells or LASCPC-01) in different concentration gradients of cisplatin, and the IC_50_ value was measured and analyzed after 48 h.

### Cell viability assays

For cell viability and IC_50_ value of mouse NEPC cells, 5 × 10^3^ cells were seeded into 96-well plates and treated with conditioned medium or recombined CCL5 under cisplatin treatment for 48 h and 10 µL CCK-8 reagent (A311-1, Vazyme, China) was added to each well. Then the plates were incubated in the dark incubator for 1.5 h. The absorbance values were measured at 450 nm.

For cell viability and IC_50_ value of LASCPC-01 cells, 1 × 10^4^ cells were seeded into 96-well plates and treated with conditioned medium or recombined CCL5 under cisplatin treatment for 48 h before performing the assay with CellTiter-Glo luminescent assay (G7570, Promega, USA) according to the manufacturer’s guidance. The IC_50_ values were determined using non-linear regression curve fit model in GraphPad Prism 8.0, following the manufacturer’s instructions. Briefly, under the dose-response–inhibition module, log [cisplatin concentration] vs. response – variable slope was utilized to determine the IC_50_. For drug response assays, mouse NEPC cells and LASCPC-01 cells were treated with cisplatin at concentrations ranging from 0.1 to 30 µg/mL and from 0.03 to 10 µg/mL, respectively, and cell viability was assessed after 48 h. Cell viability assays were performed with at least three independent biological replicates.

### Plasmids, lentiviral production, and siRNA

The pLVX304-ARRB1-V5 was purchased from the Core Facility of Basic Medical Sciences, Shanghai Jiao Tong University School of Medicine. The pCMV HA-p85α and pCMV HA-p85β were kindly provided by Dr. Yujun Hao [21]. The pIP-flag-CCR5-CMV was constructed by inserting the human CCR5 sequence into the pIP-flag-CMV vector. Short-hairpin RNA (shRNA) plasmids targeting human CCL5, CCR1, CCR3, CCR4, CCR5, β-arrestin1, β-arrestin2, p85α, p85β, and p110α were constructed using the pLKO.1 vector. ShRNA plasmids targeting mouse CCL5, CCR1, CCR3, CCR4, CCR5 were constructed using the pLKO.1 vector. All plasmids were identified by DNA Sanger sequencing.

Lentivirus was prepared using a three-plasmid packing system. In brief, expression plasmids were co-transfected with psPAX2 and pMD2.G into HEK293T cells to generate lentivirus. Viral supernatants were collected at 48 and 72 h post-transfection and filtered through a 0.45 μm membrane. Stable cell lines were selected out in 1–2 µg/mL puromycin or 5–10 µg/mL blasticidin S for 2 weeks.

For siRNA experiments, 0.5 µmol of siRNA was diluted in Opti-MEM medium with Lipofectamine 3000 kit reagent (L3000075, Invitrogen, USA) for 15 min at room temperature and then added to the cells. After 6 h, the medium was replaced by fresh medium. The shRNA and siRNA sequences used are listed in Table S3.

### RNA sequencing and transcriptome analysis

Transcriptome sequencing and subsequent analysis were carried out by OE Biotech Co., Ltd. (Shanghai, China). This has been described in detail in our previous publication [12]. The FPKM (Fragments Per Kilobase of transcript per Million mapped reads) method was applied to normalize gene expression levels for sequencing depth and gene length. Differentially expressed genes between the two groups were identified using the limma package v3.46.0 in R v4.0.5. A cutoff of *P* < 0.05 with a log2FoldChange > 0.58 or < − 0.58 was applied. Volcano plots and heatmap were generated using the ggplot2 package and pheatmap package. Gene set enrichment analysis (GSEA) was conducted using the GSEA function of the ClusterProfiler package v3.18.1 or GSEA software v4.2.3 to examine biological differences. The SENESCENCE gene set was derived from the previously published study [22], which provided a curated list of genes associated with cellular senescence. Additional gene sets, including those from Gene Ontology and Kyoto Encyclopedia of Genes and Genomes, adopted from the Molecular Signatures Database, were also utilized for enrichment analysis.

### Animals

All animal experiments in this study were conducted in accordance with the ethical regulations of Ren Ji Hospital and housed in a specific pathogen-free facility. The experimental protocols were approved by the Ren Ji Hospital Laboratory Animal Use and Care Committee. Tg(Pbsn-cre)4Prb/J (026662), B6.129P2-Trp53^tm1Brn^/J (008462), Rb1^tm2Brn^/J (026563), and B6.129S4-Pten^tm1Hwu^/J mice (006440) were purchased from Jackson Laboratory to generate Pb-Cre4: *Pten*^f/f^; *Trp53*^f/f^; *Rb1*^f/f^ mice. The genotyping of mice has been previously described in our earlier study [12]. Castration was initiated under anesthesia with 1% isoflurane at 8 weeks. The surgery was conducted on a heating pad, and mice were monitored until they fully recovered from anesthesia. Pb-Cre4: *Pten*^f/f^; *Trp53*^f/f^; *Rb1*^f/f^ mice were intraperitoneally injected with PBS, maraviroc (20 mg/kg), cisplatin (1 mg/kg), or a combination of maraviroc and cisplatin. B6.129P2-Ccl5^tm1Hso^/J (005090) mice were purchased from Jackson Laboratory. Genotyping results of mice were presented in Fig. S6A. All experimental mice involved in this study were male. One million mouse NEPC cells were mixed into a 50% Matrigel suspension (356237, Corning, USA) and injected subcutaneously into flanks of male B6.129P2-Ccl5^tm1Hso^/J mice (*n* = 3) or C57BL/6 wildtype mice at 8 weeks. For B6.129P2-Ccl5^tm1Hso^/J mice, cisplatin (5 mg/kg) or PBS was given by intraperitoneal injection once a week for 4 weeks. Tumor measurements began one week after injection when the tumors became palpable (calculated as Volume = 0.52 × Length × Width²).

For xenograft experiments, all male BALB/c nude mice used in this study were supplied by the animal laboratory of Ren Ji Hospital, as authorized by the Shanghai Science and Technology Commission. Measures were taken to minimize animal distress. According to institutional policies on tumor production, the maximum permissible tumor volume is 2000mm³, and this limit was not exceeded in any of our experiments. Once the tumors became palpable, the mice were randomly assigned to different experimental groups. For the injection experiment, 5 × 10^6^ LASCPC-01 cells were subcutaneously injected alone or co-injected with 5 × 10^6^ human CAFs (mixed into a 50% Matrigel suspension) into the right flank of male nude mice (*n* = 5) at 6 weeks. For drug treatment experiments, cisplatin (5 mg/kg) was given by intraperitoneal injection once a week for 4 weeks. Maraviroc (20 mg/kg) was given by intraperitoneal injection 3 times a week for 4 weeks.

### Patient-derived xenografts and organoids

NEPC patient-derived xenografts were established using male BALB/c nude mice. Briefly, human NEPC xenografted tumors were chopped, minced into 3 mm fragments, and subcutaneously transported into the BALB/c nude mice. Cisplatin (5 mg/kg) or PBS was given by intraperitoneal injection once a week for 4 weeks. The tumor tissues were used to verify changes in CCL5 expression and to identify the source cells of CCL5. Two NEPC patient-derived organoid models (NEPC PDO#1 and NEPC PDO#2) were established from the patient-derived xenografts without sorting, and retaining CAFs. The clinical characteristics of the corresponding patients are summarized in Table S1. The NEPC patient-derived organoids were treated with PBS, cisplatin (0.5 µg/mL), maraviroc (1µM), or cisplatin plus maraviroc. The viability of organoids was assessed using the CellTiter-Glo luminescent cell viability assay (G7570, Promega, USA), following the manufacturer’s instructions. The organoid culture was performed according to the methods described by Chua’s group [23]. Organoid viability assays were performed with at least three independent biological replicates.

### Statistical analysis

Statistical analyses were performed using GraphPad Prism (V8.0). Data are presented as mean ± standard deviation (SD). A significance level of *p* < 0.05 was considered statistically significant. A two-tailed Student’s *t*-test was performed for all comparisons between two groups of independent datasets. For multiple comparisons (more than two groups), one-way analysis of variance (ANOVA) was performed, followed by Tukey’s multiple comparisons test as a post hoc analysis. Sample sizes were determined based on preliminary data or effect sizes reported in prior studies, ensuring > 80% power to detect biologically relevant differences. All experiments included at least three independent biological replicates (with details in the figure legends), and representative images were shown.

## Results

### Cisplatin increases CCL5 expression and secretion in cancer-associated fibroblasts

**Fig. 1:**
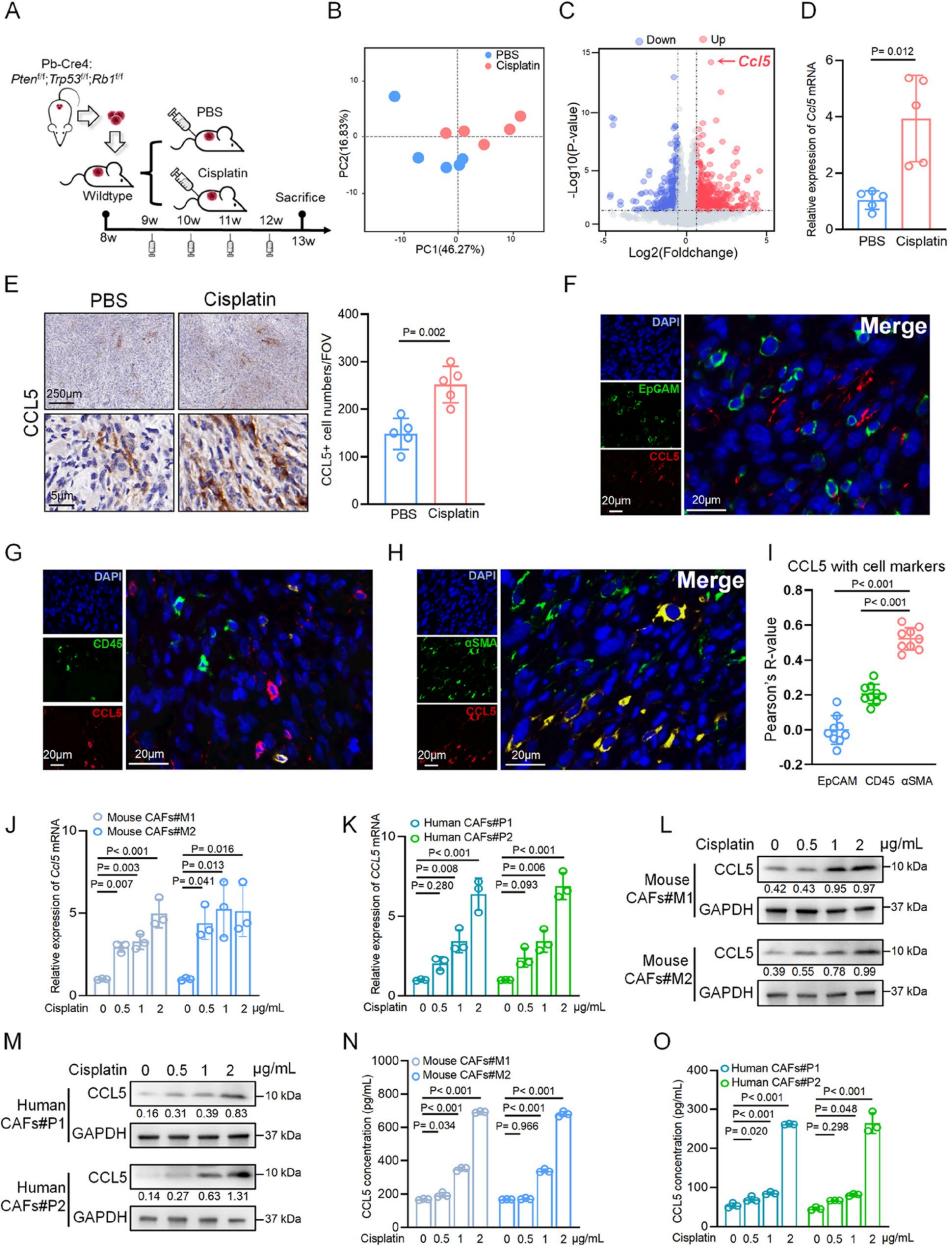
Cisplatin increases CCL5 expression and secretion in cancer-associated fibroblasts. **A** Schematic illustration showing the treatment of cisplatin or PBS in NEPC allografted mouse model. **B** Principal component analysis of transcriptomic data from allograft tumors treated with cisplatin or PBS. Each dot represents a sample that is colored on the basis of treatment (blue, PBS; red, cisplatin). **C** Volcano plots showing differentially expressed genes in the cisplatin group versus the PBS group (fold change > 2; adjusted p-value < 0.05). **D** QPCR showing relative mRNA expression of *Ccl5* in allograft tumors treated with cisplatin or PBS. **E** Representative immunohistochemistry images (left) and quantification (right) of CCL5 in allograft tumors treated with cisplatin or PBS. Scale bars, above, 250 μm; below, 5 μm. **F**-**H** Representative immunofluorescence images of CCL5 co-staining with EpCAM (**F**), CD45 (**G**), or αSMA (**H**) in allograft tumors treated with cisplatin. **I** Quantification of CCL5 co-staining with EpCAM, CD45, or αSMA. The correlation was expressed by Pearson’s R-value. **J**, **K** QPCR showing relative mRNA expression of CCL5 in mouse CAFs (**J**) and human CAFs (**K**) treated with cisplatin in indicated concentrations. **L**, **M** Western blot showing protein expression of CCL5 in mouse CAFs (**L**) and human CAFs (**M**) treated with cisplatin in indicated concentrations. **N**, **O** ELISA assays showing the concentration of CCL5 in the supernatant of mouse CAFs (**N**) and human CAFs (**O**). Both of the CAFs were treated with cisplatin in indicated concentrations. Data presented as mean and error bars report standard deviation (**D**, **E**, **I**, **J**, **K**, **N**, and **O**). Statistical significance was determined by two-tailed unpaired Student’s *t*-test (**D** and **E**) or one-way analysis of variance (ANOVA) with Dunnett’s multiple comparisons (**I**, **J**, **K**, **N**, and **O**). CAFs#M1 and CAFs#M2 are two independently mouse CAF strains isolated from NEPC mice (**J**, **L**, and **N**). CAFs#P1 and CAFs#P2 are two independently human CAF strains isolated from NEPC patients (**K**, **M**, and **O**). The protein levels were normalized to the GAPDH using ImageJ. Western blot experiments were repeated three times independently, with similar results (**L** and **M**)

To assess the chemoresistant property of neuroendocrine prostate cancer, we generated an allograft model using tumor tissues from a well-accepted NEPC mouse model (Pb-Cre4: *Pten*^f/f^; *Trp53*^f/f^; *Rb1*^f/f^) [24], which allowed us to synchronize tumor onset and monitor the response to cisplatin treatment (Fig. 1A). These allograft tumors consistently exhibited a highly aggressive and neuroendocrine phenotype, similar to parental tumors (Figs. S1A and B). Once the tumors were palpable, mice were randomized to receive either cisplatin treatment or PBS control once a week for four cycles, simulating a clinical regimen. When tumors in the PBS group reached the ethical endpoint, we collected all the tumors and performed RNA sequencing to investigate the changes after cisplatin treatment (Fig. 1A). Principal component analysis revealed a striking transcriptional distinction between the cisplatin-treated and PBS groups (Fig. 1B). Among 505 upregulated and 339 downregulated genes identified in the cisplatin group, *Ccl5* stood out based on both the fold-change and p-value (Fig. 1C). Indeed, from the chemokine/cytokine profile analysis in response to cisplatin or PBS control, CCL5 demonstrated a high intra-group concordance and remarkable inter-group divergence (Fig. S1C). To confirm this observation, we performed qPCR and immunohistochemical staining on allograft tumors and found *Ccl5* upregulation at both the RNA and protein levels following cisplatin treatment (Figs. 1D and E). Given the challenge of obtaining post-cisplatin samples from NEPC patients, we instead used paired samples from patients with prostate adenocarcinoma who received neoadjuvant cisplatin-based combination therapy in our previous clinical study [25]. A comparison of pre- and post-treatment samples revealed an increase in CCL5 intensity and staining percentage in post-treatment samples (Fig. S1D). Together, these results demonstrate that cisplatin treatment effectively induces CCL5 expression in human and murine tumor samples.

Having established that CCL5 is upregulated in response to cisplatin treatment, we next sought to identify the specific cell type responsible for its upregulation. We performed immunofluorescence co-staining on allograft tumors with a CCL5 antibody and various cell markers. A distinct and predominant overlap between CCL5 and αSMA indicated that CAFs are the primary source of CCL5 following cisplatin treatment (Figs. 1F–I). To further confirm this, we isolated CAFs from tumor tissues of both NEPC patients (Fig. S1E) and NEPC mice, hereinafter referred to as human CAFs and mouse CAFs (Figs. S1F and G), respectively, and treated these cells with cisplatin in vitro. As shown in Figs. 1J–M, cisplatin treatment was associated with a trend toward increased CCL5 expression at both RNA and protein levels, with upregulation observed at several concentrations. ELISA assays also demonstrated that CCL5 secretion in CAF-derived supernatant increased following cisplatin treatment (Figs. 1N and O). Together, these results suggest that cisplatin treatment increases both the expression and release of CCL5 in CAFs from NEPC.

### Cisplatin predisposes CAFs to senescence via the cGAS-STING pathway

**Fig. 2:**
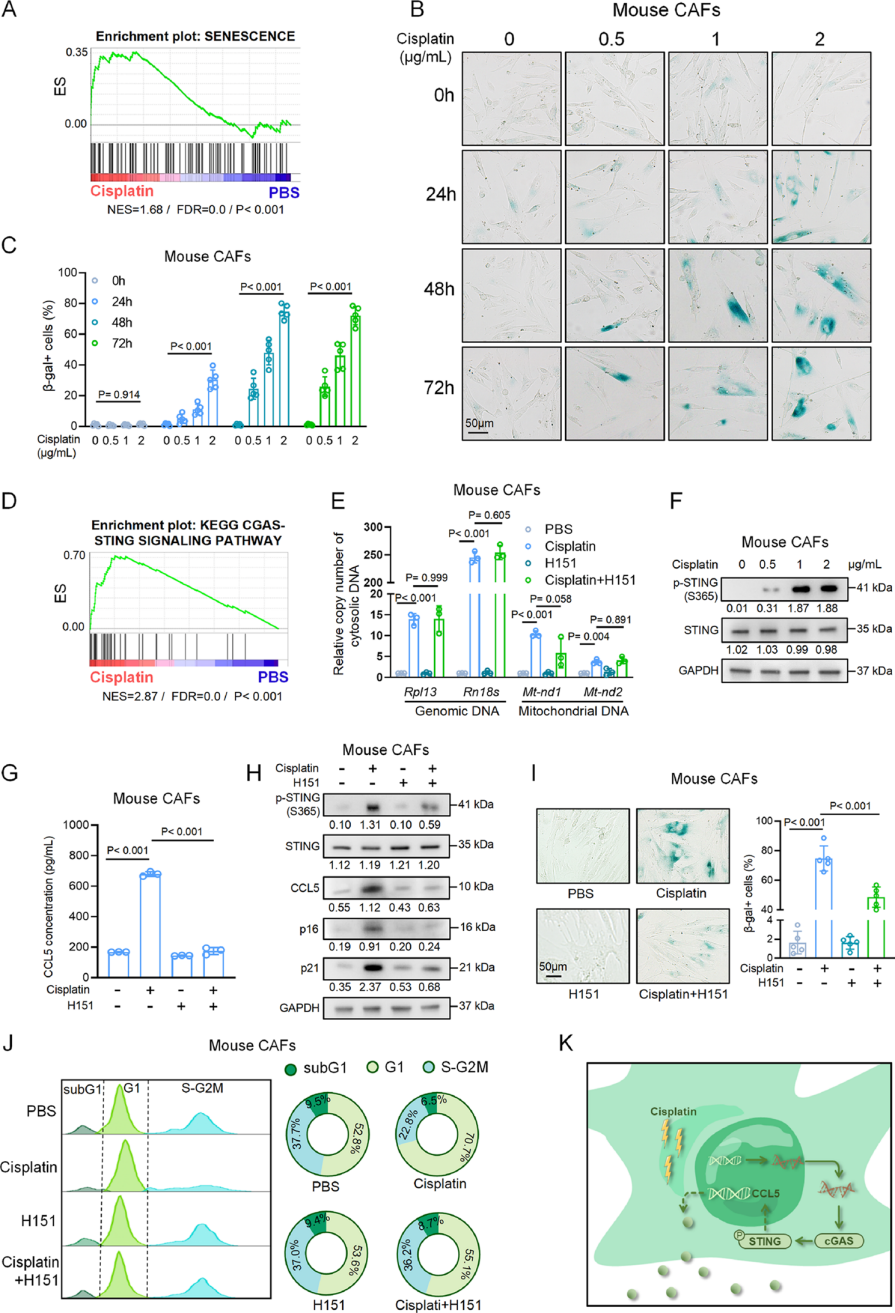
Cisplatin predisposes CAFs to senescence via the cGAS-STING pathway. **A** Gene set enrichment analysis of differentially expressed genes using senescence signature in allograft tumors treated with cisplatin versus PBS (RNA sequencing was performed on 5 independent samples per group. NES: normalized enrichment score; FDR: false discovery rate). **B** Representative β-gal staining images of mouse CAFs treated with cisplatin in indicated concentrations and times. Scale bar, 50 μm. **C** Quantification of the percentage of β-gal–positive mouse CAFs treated with cisplatin in indicated concentrations and times (*n* = 5 per group). **D** Gene set enrichment analysis of differentially expressed genes using cGAS-STING signature in allograft tumors treated with cisplatin versus PBS (RNA sequencing was performed on 5 independent samples per group. NES: normalized enrichment score; FDR: false discovery rate). **E** QPCR analysis of genomic DNA (*Rpl13* and *Rn18s*) and mitochondrial DNA (*Mt-nd1* and *Mt-nd2*) in mouse CAFs treated with PBS, cisplatin (2 µg/mL), H151 (1µM), or cisplatin plus H151. **F** Western blot showing phosphorylated and total STING levels in mouse CAFs treated with cisplatin for 48 h in indicated concentrations. **G** ELISA assay showing the concentration of CCL5 in the supernatant of mouse CAFs treated with PBS, cisplatin (2 µg/mL), H151 (1µM), or cisplatin plus H151. **H **Western blot showing expression of indicated proteins in mouse CAFs treated with PBS, cisplatin (2 µg/mL), H151 (1µM), or cisplatin plus H151. **I** Representative β-gal staining images (left) and quantification (right) of mouse CAFs treated with PBS, cisplatin (2 µg/mL), H151 (1µM), or cisplatin plus H151. **J** Cell cycle distribution by flow cytometry of mouse CAFs treated with PBS, cisplatin (2 µg/mL), H151 (1µM), or cisplatin plus H151. **K** Schematic illustration showing that cisplatin predisposed CAFs to senescence through the cGAS-STING pathway and upregulated CCL5 secretion. Data presented as mean and error bars report standard deviation (**C**, **E**, **G**, and **I**). Statistical significance was determined by ANOVA with Dunnett’s multiple comparisons (**C**, **E**,** G**, **I**, and **J**). The protein levels were normalized to the GAPDH using ImageJ. Western blot experiments were repeated three times independently, with similar results (**F** and **H**)

We next sought to investigate the upstream signaling pathway responsible for the increased CCL5 expression following cisplatin treatment. Given that CCL5 is a key senescence-associated secretory protein and previous studies have linked cisplatin to senescence-like phenotype [26], we hypothesized that cisplatin induces senescence in CAFs, resulting in increased CCL5 expression. Gene Set Enrichment Analysis of the RNA sequencing data from allograft tumors revealed enrichment of the senescence pathway in cisplatin-treated tumors (Fig. 2A). To confirm this, we performed the β-gal staining on CAFs and observed a time- and concentration-dependent increase in β-gal positivity following cisplatin treatment (Figs. 2B and C). In contrast, epithelial cells isolated from NEPC mice, hereinafter referred to as mouse NEPC cells (Figs. S2A and B), did not exhibit an increase in β-gal positivity, even treated with high cisplatin concentrations (2 µg/mL), which induced cell death instead (Figs. S2C and D). These results indicate that cisplatin preferentially induces senescence in CAFs rather than in tumor cells.

We next investigated the upstream signaling pathways driving CCL5 expression in senescent CAFs. Based on several lines of evidence, we hypothesized that the cGAS-STING pathway plays a central role in regulating CCL5 expression in cisplatin-induced senescent CAFs: (1) the activated cGAS-STING pathway regulates CCL5 transcription mainly via TBK1-mediated activation of IRF3 and NF-κB [27, 28]; (2) cGAS senses cytosolic DNA fragments in senescent cells, a phenotype induced by cisplatin [29, 30]; and (3) our Gene Set Enrichment Analysis demonstrated significant enrichment of the cGAS-STING pathway in cisplatin-treated allograft tumors (Fig. 2D). To test this speculation, we assessed cytosolic DNA release in CAFs and showed that cisplatin dramatically stimulated the release of both genomic DNA and mitochondrial DNA into the cytosol (Figs. 2E and S2E). This treatment also enhanced STING phosphorylation and increased CCL5 secretion in both human and mouse CAFs (Figs. 2E– G and S2E–G). Furthermore, cisplatin treatment induced senescence in CAFs, as demonstrated by increased p16 (*Cdkn2a*) and p21 (*Cdkn1a*), β-gal staining, and G1 arrest (Figs. 2H–J, S2F, H, and I). The STING inhibitor H151 [31] effectively blocked the cisplatin-induced CCL5 secretion and decreased cisplatin-induced senescent phenotype (Figs. 2G–J and S2F–I), although it did not attenuate the release of cytosolic DNA (Figs. 2E and S2E). Collectively, these results establish that cisplatin treatment drives cytosolic DNA release, activates the cGAS-STING pathway, and induces senescence in CAFs, resulting in CCL5 expression and secretion (Fig. 2K).

### CAF-derived CCL5 mediates chemotherapy resistance of cancer cells

**Fig. 3:**
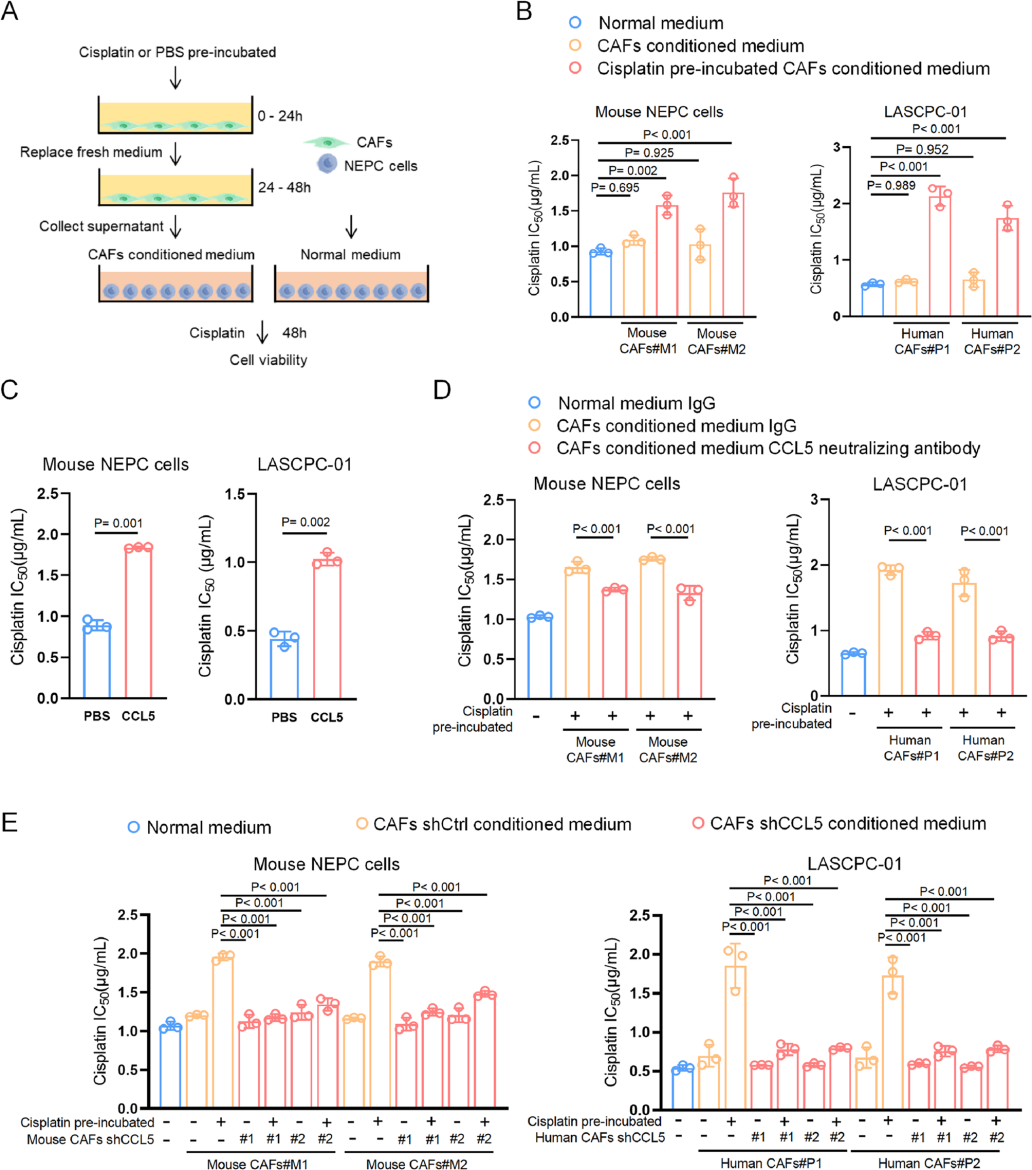
CAF-derived CCL5 mediates chemotherapy resistance of cancer cells. **A** Schematic illustration showing that CAFs were pre-incubated with cisplatin or PBS and then supernatants were collected as a conditioned medium. NEPC cells were treated with normal medium or conditioned medium from CAFs and used for evaluation of cell response to cisplatin. **B** IC_50_ values of cisplatin in NEPC cells cultured with conditioned medium from human or mouse CAFs which were pre-incubated with cisplatin or PBS. **C** IC_50_ values of cisplatin in NEPC cells treated with recombinant CCL5 protein (100ng/mL) or PBS. **D** IC_50_ values of cisplatin in NEPC cells cultured with conditioned medium from CAFs together with CCL5 neutralizing antibody or IgG control. **E** IC_50_ values of cisplatin in NEPC cells cultured with conditioned medium from CAFs with control shRNA or two independent shRNAs targeting CCL5. Data presented as mean and error bars report standard deviation (**B**–**E**). Statistical significance was determined by one-way analysis of variance (ANOVA) with Dunnett’s multiple comparisons (**B**–**E**). CAFs#M1 and CAFs#M2 are two independently mouse CAF strains isolated from NEPC mice (Pb-Cre4: *Pten*^f/f^; *Trp53*^f/f^; *Rb1*^f/f^) (**B**, **D**, and **E**). CAFs#P1 and CAFs#P2 are two independently human CAF strains isolated from NEPC patients (**B**, **D**, and **E**). Two independent shRNA sequences targeting CCL5 mRNA were labeled as shCCL5#1 and shCCL5#2 (**E**)

To investigate whether CAF-derived CCL5 mediates the protective effect of tumor cells against cisplatin treatment, we first co-cultured tumor cells with a conditioned medium obtained from CAFs and tested the cells’ response to cisplatin treatment (Fig. 3A). As reflected by IC_50_ values and time-dependent cell viability, medium from cisplatin-treated CAFs greatly boosted tumor cell survival, while treatment-naïve CAFs slightly enhanced it, indicating the essential role of cisplatin pre-treated CAF-derived factors in chemoresistance (Figs. 3B, S3A and B).

To determine whether CCL5 is specifically responsible for this effect, we supplemented recombinant CCL5 and assessed tumor cell responses to cisplatin. CCL5 dramatically increased the IC_50_ value by approximately 2-fold in both human and mouse tumor cells (Fig. 3C). Conversely, treatment with a CCL5-neutralizing antibody effectively abrogated the resistance mediated by CAF-derived conditioned medium (Fig. 3D). We further knocked down CCL5 in both human and mouse CAFs using two independent shRNA sequences (Figs. S3C–H). Under cisplatin treatment, CCL5-deficient CAF-derived conditioned medium did not enhance cisplatin resistance, while wildtype CAF-derived medium did (Fig. 3E). These results establish that CAF-derived CCL5 effectively promotes cisplatin resistance in tumor cells.

### The CCL5/CCR5 axis protects cancer cells against cisplatin-induced DNA damage and apoptosis

**Fig. 4:**
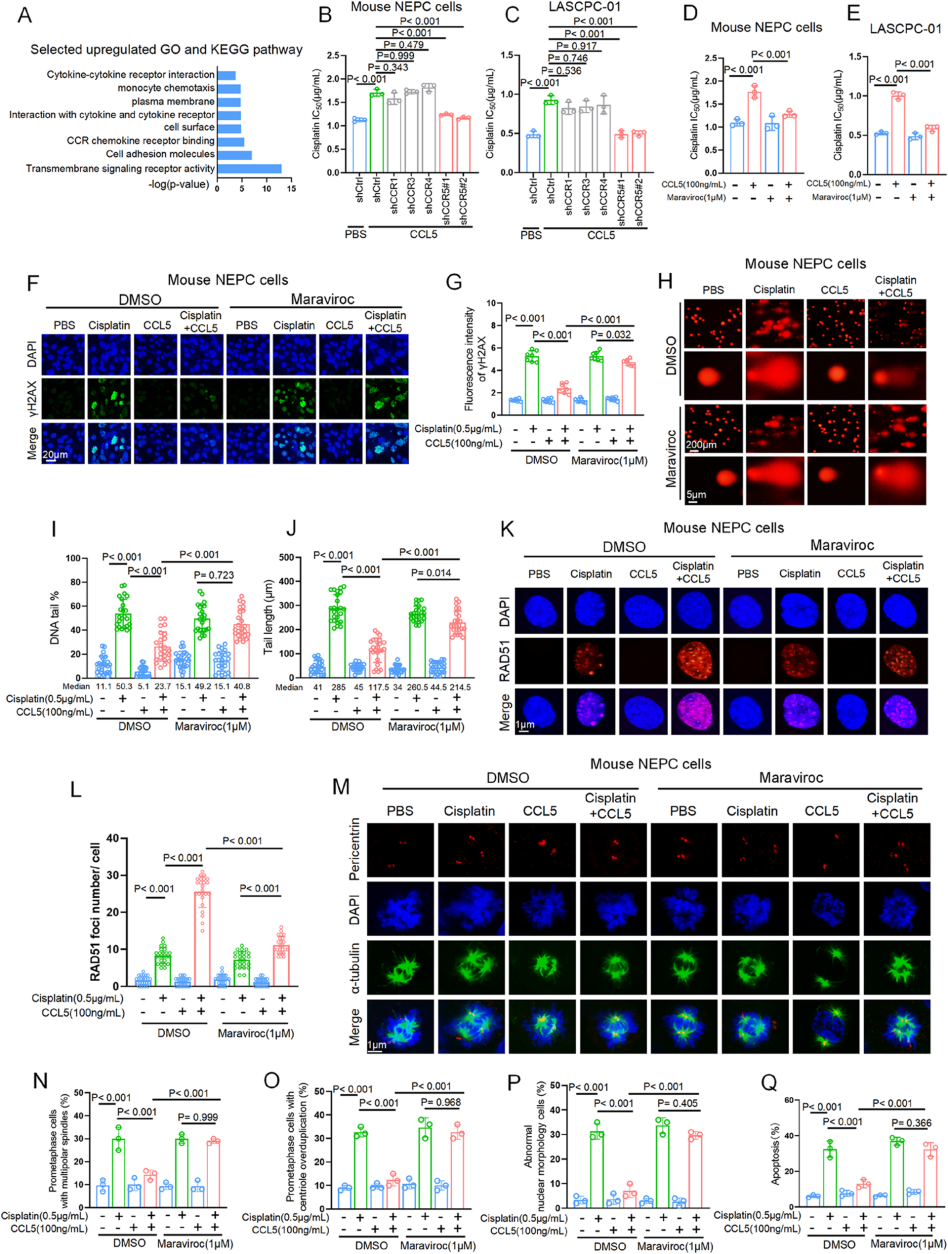
The CCL5-CCR5 axis protects cancer cells against cisplatin-induced DNA damage and apoptosis. **A** Enriched Gene Ontology (GO) and Kyoto Encyclopedia of Genes and Genomes (KEGG) gene sets in genes upregulated in allograft tumors treated with cisplatin versus PBS. **B** IC_50_ values of cisplatin in mouse NEPC with shCtrl, shCCR1, shCCR3, shCCR4, or shCCR5 treated with recombinant CCL5 protein (100ng/mL). **C** IC_50_ values of cisplatin in LASCPC-01 with shCtrl, shCCR1, shCCR3, shCCR4, or shCCR5 treated with recombinant CCL5 protein (100ng/mL). **D** IC_50_ values of cisplatin in mouse NEPC cells with indicated treatments. **E** IC_50_ values of cisplatin in LASCPC-01 with indicated treatments. **F**, **G** Representative immunofluorescence images (**F**) and quantification (**G**) for γH2AX in mouse NEPC cells with indicated treatments (*n* = 7 per group; blue, DAPI; green, γH2AX). **H** Representative images of alkaline comet assay in mouse NEPC cells with indicated treatments. **I**, **J** Quantification of alkaline comet assay in mouse NEPC cells with indicated treatments (*n* = 24 per group). Median values for each group are presented below the x-axis. **K**, **L** Representative images (**K**) and quantification (**L**) of RAD51 foci in mouse NEPC cells with indicated treatments (*n* = 24 per group). **M** Representative images of α-tubulin, pericentrin, and DAPI staining in mouse NEPC cells with indicated treatments. **N**–**P **Quantification of multipolar spindles (**N**), centrosome amplification (**O**), and abnormal nuclear morphology (**P**) in mouse NEPC cells with indicated treatments. **Q** Quantification of the percentage of apoptotic cells by Annexin V/propidium iodide staining in mouse NEPC cells with indicated treatments. Data presented as mean and error bars report standard deviation (**B**–**E**, **G**, **I**, **J**, **L**, and **N**–**Q**). Statistical significance was determined by ANOVA with Dunnett’s multiple comparisons (**B**–**E**, **G**, **I**, **J**, **L**, and **N**–**Q**). Two independent shRNA sequences targeting CCR5 mRNA were labeled as shCCR5#1 and shCCR5#2 (**B** and **C**)

To investigate how CAF-derived CCL5 modulated cisplatin resistance of NEPC cells, we re-analyzed the transcriptome data from allograft tumors and revealed a significant enrichment of pathways associated with transmembrane receptors signaling in the cisplatin-treated group (Fig. 4A). CCL5 primarily exerts its effects by binding and activating cell membrane receptors, including CCR1, CCR3, CCR4, and CCR5 [32–34]. To identify the receptor responsible for cisplatin resistance, we knocked down each of these receptors in both human and mouse NEPC cells and assessed their response to cisplatin (Figs. S4A–D). In the absence of CCL5, all receptor-knockdown cells responded to cisplatin similarly to control cells (Figs. S4E and S4F). However, upon the addition of recombinant CCL5, only CCR5-deficient cells failed to exhibit the increased cisplatin resistance observed in control cells, as measured by IC_50_ (Figs. 4B and C). We further tested whether pharmacological inhibition of CCR5 with maraviroc [35] could abrogate CCL5-mediated cisplatin resistance. While maraviroc alone had minimal impact on cell viability in the presence or absence of cisplatin (Figs. S4G and H), it effectively inhibited CCL5-mediated cisplatin resistance (Figs. 4D and E, S4G and H). These results suggest that CCR5 is the key receptor mediating the protective effects of CCL5.

Given that cisplatin functions primarily by targeting DNA, we measured DNA damage by detecting γH2AX foci, a sensitive reporter of DNA double-strand breaks. As shown in Figs. 4F and G, treatment with recombinant CCL5 inhibited cisplatin-induced γH2AX foci formation, indicating reduced DNA double-strand breaks. Importantly, this inhibition was reversed by maraviroc treatment. An alkaline comet assay further confirmed that CCL5 protected NEPC cells from cisplatin-induced DNA breaks, as indicated by reduced tail length and intensity of tail moments. Again, this protective effect was abolished by maraviroc treatment (Figs. 4H–J). We next examined the DNA repair capacity by RAD51 staining in mouse NEPC cells and showed that while CCL5 increased RAD51 foci formation at the presence of cisplatin, maraviroc effectively attenuated the effect of CCL5 (Figs. 4K and L). We also detected the ubiquitination of PCNA, another DNA repair biomarker which suggested translesion synthesis recruitment to tolerate DNA damage. However, despite cisplatin dramatically induced PCNA ubiquitination, CCL5 failed to attenuate this effect, indicating that CCL5 does not act through the translesion synthesis pathway (Fig. S4I). Given that cisplatin-induced DNA damage may triggers mitotic catastrophe in tumor cells, we asked whether CCL5 may protect cells from this process. We performed immunofluorescent staining on mouse NEPC cells and examined microtubules (α-tubulin), centrosome (pericentrin), and nuclei (DAPI) to detect any alteration in microtubule organization and nuclear morphology. As shown in Figs. 4M–P, cisplatin treatment dramatically induced mitotic catastrophe as demonstrated by multipolar spindle formation, centriole overduplication, and abnormal nuclear morphology (multinucleation, micronucleation, nuclear condensation, and nuclear fragmentation). Notably, while CCL5 attenuated the effect of cisplatin on mitotic catastrophe, maraviroc counteracted this effect of CCL5 and resensitized tumor cells to cisplatin. To further examine the effect of CCL5 on cisplatin-induced apoptosis, we stained mouse NEPC cells with Annexin V/propidium iodide and demonstrated that CCL5 effectively inhibited cell apoptosis in response to cisplatin (Figs. 4Q and S4J). Importantly, the combination of cisplatin and maraviroc restored apoptosis to levels comparable to cisplatin treatment alone (Figs. 4Q and S4J). Together, these findings highlight the critical role of the CCL5/CCR5 pathway in protecting NEPC cells from cisplatin-induced DNA damage and apoptosis.

### CCL5 mediates cisplatin resistance through the PI3K/AKT pathway

**Fig. 5:**
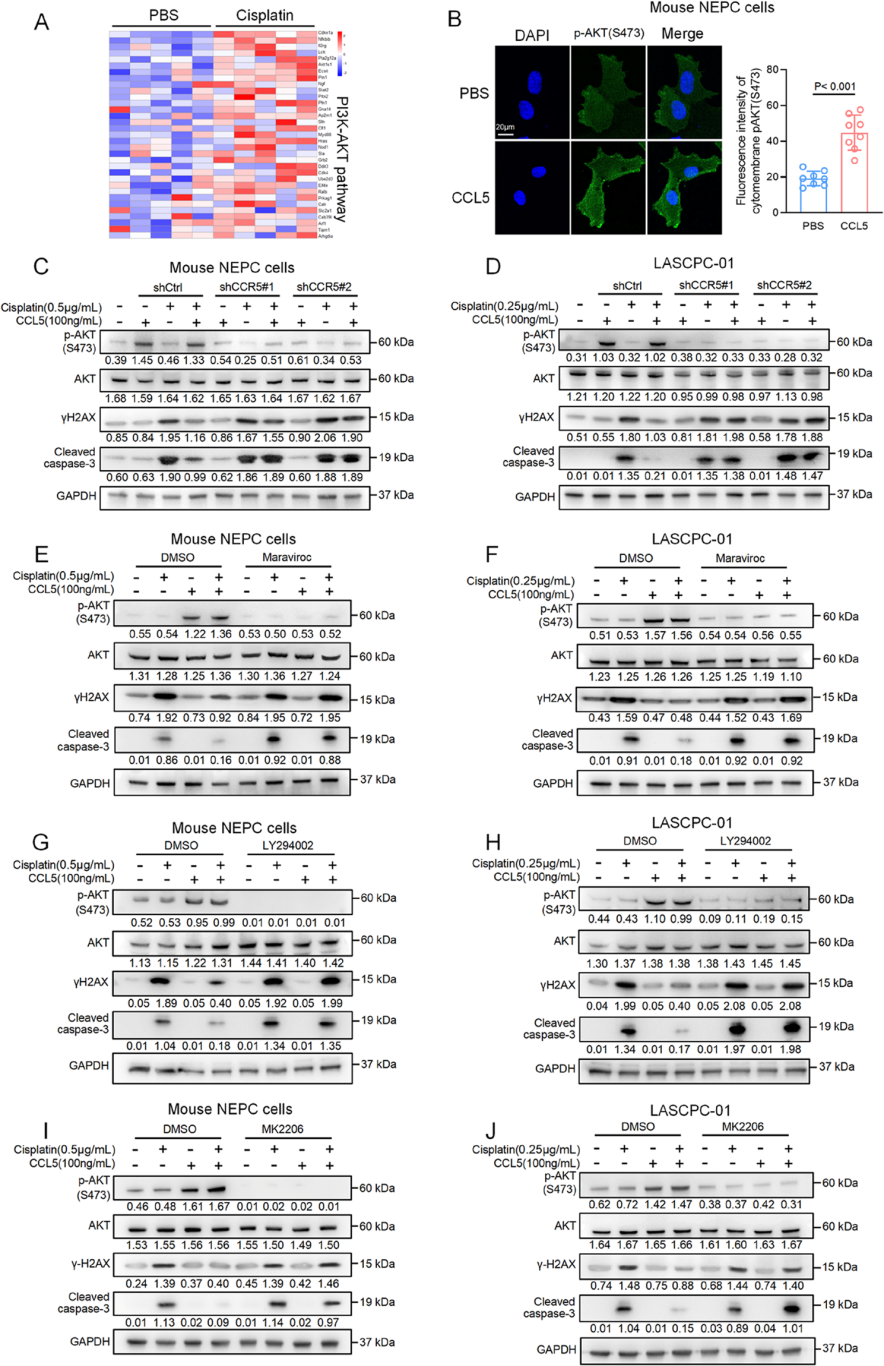
CCL5 mediates cisplatin resistance through the PI3K/AKT pathway. **A** Heatmap showing the expression of the PI3K/AKT pathway-related genes in allograft tumors treated with cisplatin or PBS. **B** Representative immunofluorescence images (left) and quantification (right) of phosphorylated AKT(S473) in mouse NEPC cells treated with CCL5 or PBS. (*n* = 8 per group). Scale bar, 20 μm. **C**, **D** Western blot showing protein expression of phosphorylated AKT(S473), AKT, γH2AX, and cleaved caspase-3 in mouse NEPC cells (**C**) and LASCPC-01 (**D**) with control shRNA or two independent shRNA targeting CCR5, in combination with the indicated treatments. **E**, **F** Western blot showing protein expression of phosphorylated AKT(S473), AKT, γH2AX, and cleaved caspase-3 in mouse NEPC cells (**E**) and LASCPC-01 (**F**) treated with DMSO or maraviroc targeting CCR5, in combination with the indicated treatments. **G**, **H** Western blot showing protein expression of phosphorylated AKT(S473), AKT, γH2AX, and cleaved caspase-3 in mouse NEPC cells (**G**) and LASCPC-01 (**H**) treated with DMSO or LY294002 targeting PI3K, in combination with the indicated treatments. **I**, **J** Western blot showing protein expression of phosphorylated AKT(S473), AKT, γH2AX, and cleaved caspase-3 in mouse NEPC cells (**I**) and LASCPC-01 (**J**) treated with DMSO or MK2206 targeting AKT, in combination with the indicated treatments. Data presented as mean and error bars report standard deviation (**B**). Statistical significance was determined by two-tailed unpaired Student’s *t*-test (**B**). Two independent shRNA sequences targeting CCR5 mRNA were labeled as shCCR5#1 and shCCR5#2 (**C** and **D**). The protein levels were normalized to the GAPDH using ImageJ. Western blot experiments were repeated three times independently, with similar results (**C**–**J**)

Next, we investigated the downstream signaling of the CCL5/CCR5 pathway, which mediates cisplatin resistance in NEPC cells. Transcriptome analysis of allograft tumors revealed a remarkable upregulation of the PI3K/AKT pathway in cisplatin-treated tumors compared with the PBS-treated tumors (Fig. 5A). Given AKT’s established role in cisplatin resistance [36, 37] and its link to CCR signaling [38, 39], we hypothesized that CCL5 mediates resistance through the CCR5/PI3K/AKT cascade. Immunofluorescent staining showed that CCL5 treatment induced the recruitment of phosphorylated AKT to the plasma membrane in NEPC cells (Fig. 5B). In both human and mouse NEPC cells, CCL5 alone was sufficient to increase the phosphorylation of AKT, but had no effect on ERK, JNK, or STAT3, highlighting the predominant role of the PI3K/AKT pathway in response to CCL5 (Fig. 5C and D, and S5A). The activation of AKT by CCL5 suppressed cisplatin-induced γH2AX and cleaved caspase-3 expression, consistent with its protective effects against DNA damage and apoptosis. In contrast, in CCR5-deficient cells, CCL5 failed to activate AKT or prevent γH2AX and cleaved caspase-3 expression, underscoring the essential role of CCR5 in this signaling cascade (Figs. 5C and D). To further elucidate the transduction pathway triggered by CCL5, we treated both human and mouse NEPC cells with a panel of inhibitors targeting CCR5 (maraviroc), PI3K (LY294002), and AKT (MK2206) (Fig. S5B) [35, 40, 41]. All these inhibitors effectively blocked CCL5-mediated AKT activation (Figs. 5E–J). Furthermore, they restored cisplatin-induced DNA damage and cell apoptosis, as revealed by γH2AX and cleaved caspase-3 expression under CCL5 treatment (Figs. 5E–J). Collectively, these findings demonstrate that the PI3K/AKT pathway is essential for CCL5-mediated cisplatin resistance in NEPC cells.

### CCL5 induces AKT phosphorylation by promoting the formation of CCR5/β-arrestin1/p85 complex

**Fig. 6:**
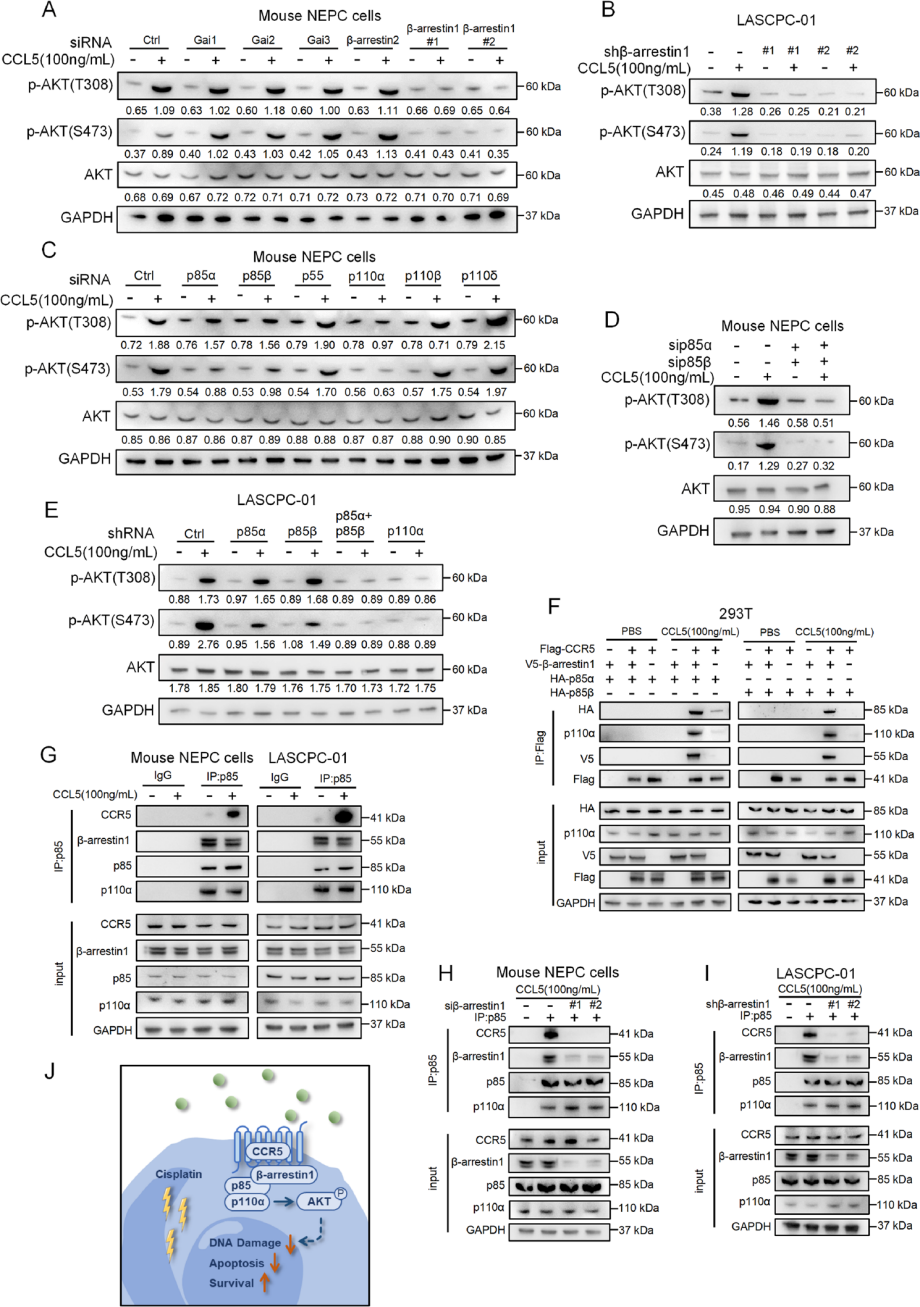
CCL5 induces AKT phosphorylation by promoting the formation of CCR5/β-arrestin1/p85 complex. **A** Western blot showing protein expression of phosphorylated AKT(T308), phosphorylated AKT(S473), and AKT in indicated mouse NEPC cells treated with CCL5 or PBS. **B** Western blot showing protein expression of phosphorylated AKT(T308), phosphorylated AKT(S473), and AKT in indicated LASCPC-01 treated with CCL5 or PBS. **C** Western blot showing protein expression of phosphorylated AKT(T308), phosphorylated AKT(S473), and AKT in indicated mouse NEPC cells treated with CCL5 or PBS. **D** Western blot showing protein expression of phosphorylated AKT(T308), phosphorylated AKT(S473), and AKT in indicated mouse NEPC cells treated with CCL5 or PBS. **E** Western blot showing protein expression of phosphorylated AKT(T308), phosphorylated AKT(S473), and AKT in indicated LASCPC-01 treated with CCL5 or PBS. **F** Co-immunoprecipitation showing the interaction between Flag-CCR5, V5–β-arrestin1, and HA-p85α/HA-p85β using cell lysates from transiently transfected HEK293T cells. **G** Co-immunoprecipitation showing the interaction between endogenous p85, CCR5, and β-arrestin1 in mouse NEPC cells (left) and LASCPC-01 (right) treated with CCL5 or PBS. **H** Co-immunoprecipitation showing the interaction between endogenous p85, CCR5, and β-arrestin1 in indicated mouse NEPC cells. **I** Co-immunoprecipitation showing the interaction between endogenous p85, CCR5, and β-arrestin1 in indicated LASCPC-01. **J** Schematic illustration showing that CCL5 facilitates the formation of the CCR5/β-arrestin1/p85 complex and activates the PI3K-AKT signaling pathway Two independent siRNA sequences targeting β-arrestin1 mRNA were labeled as siβ-arrestin1#1 and siβ-arrestin1#2 (**A** and **H**). Two independent shRNA sequences targeting β-arrestin1 mRNA were labeled as shβ-arrestin1#1 and shβ-arrestin1#2 (**B** and **I**). The protein levels were normalized to the GAPDH using ImageJ. Western blot experiments were repeated three times independently, with similar results (**A**–**I**)

We next investigated the mechanism of CCL5/CCR5-induced AKT activation. As a G protein-coupled receptor, CCR5 primarily functions by coupling extracellular stimuli—such as CCL5—to the activation of specific G proteins or β-arrestins, thereby triggering intracellular signal transduction [38, 42, 43]. Building on previous structural studies that characterized the interactions between CCR5 and β-arrestins [44] or Gi proteins [45], we knocked down β-arrestin1 (*Arrb1*), β-arrestin2 (*Arrb2*), and key catalytic components of Gi, including Gαi1 (*Gnai1*), Gαi2 (*Gnai2*), and Gαi3 (*Gnai3*), respectively (Figs. S5C–D). CCL5-mediated AKT phosphorylation was abolished only in the absence of β-arrestin1 (Fig. 6A). This finding was confirmed in a well-accepted human NEPC cell line LASCPC-01, in which β-arrestin1 ablation attenuated CCL5-induced AKT phosphorylation (Fig. 6B). To further investigate the molecular details of this signaling cascade, we knocked down each PI3K subunit in mouse NEPC cells, including p85α (*Pik3r1*), p85β (*Pik3r2*), p55 (*Pik3r3*), p110α (*Pik3ca*), p110β (*Pik3cb*), and p110δ (*Pik3cd*) (Figs. S5E–L). Knockdown of p110α or the simultaneous knockdown of p85α and p85β was sufficient to inhibit CCL5-mediated AKT phosphorylation (Figs. 6C–E). These results highlight the importance of the PI3K regulatory subunit p85 and catalytic subunit p110α in transducing CCL5 signaling. Having identified the key PI3K subunits involved, we next sought to determine whether β-arrestin1 interacts with PI3K to form a signaling complex with CCR5. Previous studies have reported an interaction between p85 and β-arrestin1 [46], leading us to hypothesize that CCR5, β-arrestin1, and p85 form a complex that transduces CCL5-induced AKT phosphorylation via p110α. To test this hypothesis, we conducted co-immunoprecipitation assays in HEK293T cells co-expressing Flag-CCR5, V5–β-arrestin1, and HA-p85α/HA-p85β. CCR5 co-immunoprecipitated with β-arrestin1 and p85α/p85β only in the presence of CCL5, which also induced AKT phosphorylation, linking the complex to downstream signaling. This interaction was abolished in the absence of β-arrestin1 (Fig. 6F). Subsequent co-immunoprecipitation assays in both human and mouse NEPC cells confirmed the formation of a CCR5/β-arrestin1/p85 complex (Fig. 6G), which was disrupted by β-arrestin1 knockdown (Figs. 6H and I). Collectively, these findings demonstrate that CCL5 promotes the formation of the CCR5/β-arrestin1/p85 complex, which is essential for AKT activation in response to CCL5 (Fig. 6J).

### Targeting the CCL5/CCR5 pathway overcomes cisplatin resistance and enhances chemotherapy efficiency

**Fig. 7:**
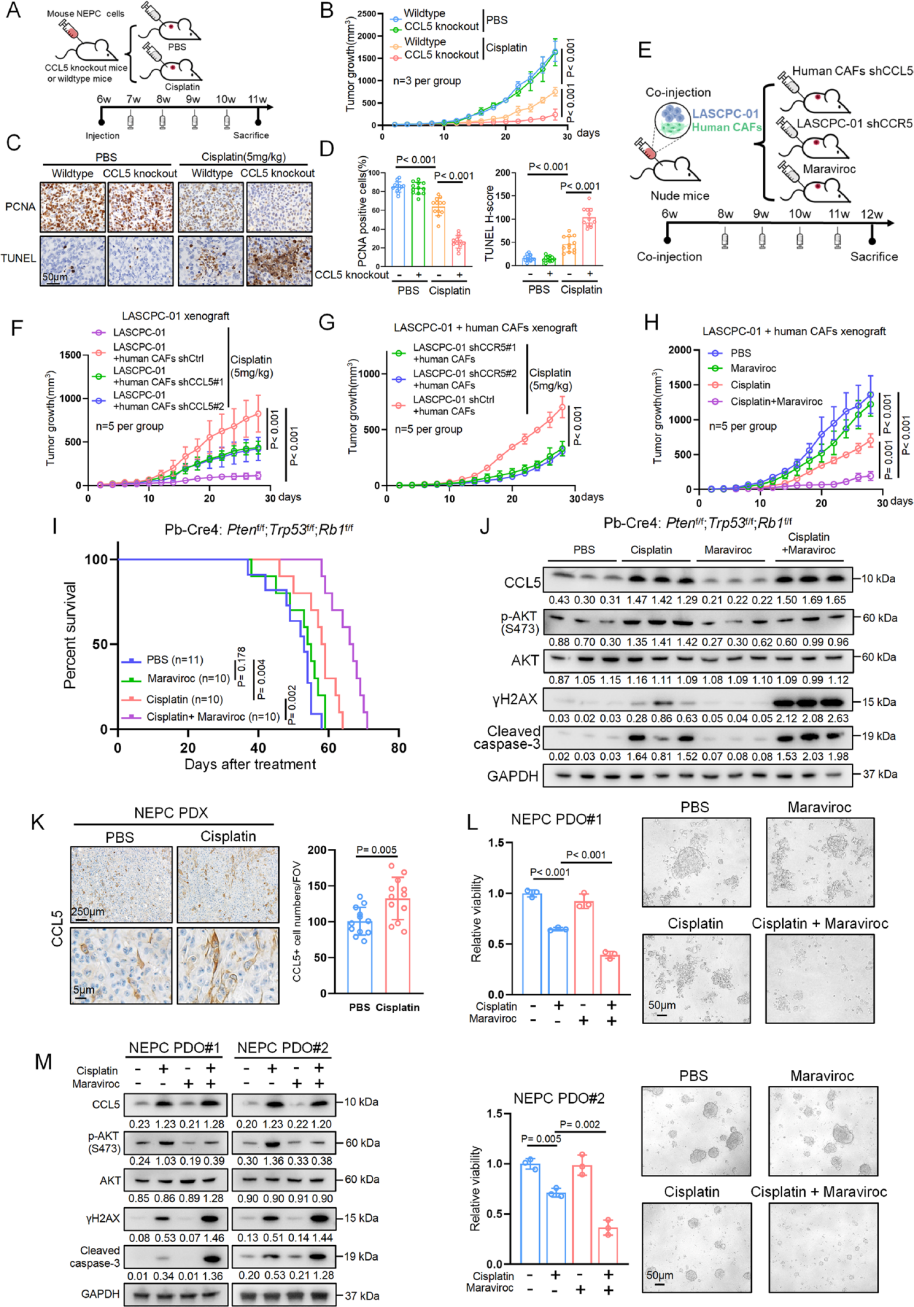
Targeting the CCL5/CCR5 pathway overcomes cisplatin resistance and enhances chemotherapy efficiency. **A **Schematic of cisplatin or PBS treatment in CCL5 knockout and wildtype mice after subcutaneous implantation. **B** Tumor growth in CCL5 knockout or wildtype mice implanted with tumor cells derived from NEPC mice, following the indicated treatments. **C**, **D** Representative immunohistochemistry images (**C**) and quantification (**D**) of PCNA and TUNEL in tumors from (**B**). Scale bar, 50 μm. **E** Schematic illustration showing co-injection model of LASCPC-01 and human CAFs in nude mice. **F** Tumor growth in nude mice bearing LASCPC-01 alone or LASCPC-01 with indicated human CAFs. **G** Tumor growth in nude mice bearing indicated LASCPC-01 with human CAFs. **H** Tumor growth in nude mice bearing LASCPC-01/human CAFs co-injection tumors with indicated treatments. **I** Kaplan-Meier survival analysis of NEPC mice treated with PBS, cisplatin, maraviroc, or cisplatin plus maraviroc. **J** Western blot showing indicated protein expression in mouse NEPC tumors treated with PBS, cisplatin, maraviroc, or cisplatin plus maraviroc. **K **Representative immunohistochemistry images (left) and quantification (right) of CCL5 in NEPC patient-derived xenograft (PDX) tumors treated with cisplatin or PBS. Scale bars, above, 250 μm; below, 5 μm. **L** Relative viabilities (left) and representative organoids images (right) of two independent NEPC patient-derived organoids (PDO#1 and PDO#2) with indicated treatments. Representative brightfield images showing growth of organoids with indicated treatments. **M** Western blot showing indicated protein expression in NEPC patient-derived organoids treated with PBS, cisplatin, maraviroc, or cisplatin plus maraviroc. Data presented as mean and error bars report standard deviation (**B**,** D**, **F**, **G**, **H**, **K**, and **L**). Statistical significance was determined by ANOVA with Dunnett’s multiple comparisons (**B**, **D**, **F**, **G**, **H**, and **L**), two-tailed unpaired Student’s *t*-test (**K**), or log-rank (Mantel-Cox) test (**I**). Two independent shRNA sequences targeting CCL5 and CCR5 were labeled as shCCL5#1/#2 (**F**) and shCCR5#1/#2 (**G**), respectively. The protein levels were normalized to the GAPDH using ImageJ. Western blot experiments were repeated three times independently, with similar results (**J** and **M**)

The above results revealed the distinct role of the CCL5/CCR5/β-arrestin1/PI3K/AKT cascade in cisplatin resistance and prompted us to evaluate the therapeutic potential of targeting this pathway in preclinical models. We first injected mouse NEPC cells into CCL5 knockout mice or their wildtype littermates (Figs. 7A and S6A). In the absence of cisplatin, tumor growth was comparable between the two groups (Fig. 7B). However, under cisplatin treatment, tumor growth was significantly reduced in CCL5 knockout recipient mice compared with wildtype controls (Fig. 7B). Histochemical analysis revealed no difference in tumor proliferation and apoptosis between the knockout and wildtype groups without cisplatin, as revealed by PCNA and TUNEL staining (Figs. 7C and D). In contrast, under cisplatin treatment, tumors from CCL5 knockout mice showed decreased proliferation and increased apoptosis compared with those from controls, highlighting the essential role of CCL5 in the tumor microenvironment during cisplatin treatment (Figs. 7C and D).

Next, we tested the effects of the CCL5/CCR5 pathway on xenograft tumor growth via tumor-stromal interactions. Nude mice were subcutaneously injected with LASCPC-01 cells alone or co-injected with human CAFs, followed by cisplatin treatment (Fig. 7E). Co-injection with CAFs significantly enhanced tumor growth compared with LASCPC-01 cells alone (Fig. 7F). This effect was attenuated when CCL5 was knocked down in CAFs, despite CCL5 ablation having no impact on cell proliferation or the epithelial-to-fibroblast ratio in xenografts (Figs. 7F, S6B and C). These findings suggest that CAF-derived CCL5 regulates tumor cell response to cisplatin. Supporting this, CCR5 knockdown in LASCPC-01 cells significantly reduced the tumor growth in xenografts of co-injection with CAFs (Figs. 7G and S6D). Maraviroc showed a similar effect as the knockdown of CCR5 on sensitizing the xenografts to cisplatin treatment, although maraviroc alone barely inhibited tumor growth (Figs. 7H and S6E). NSE, PCNA, and TUNEL staining further confirmed these results: while maraviroc alone had mild effect, its combination with cisplatin suppressed neuroendocrine phenotype, tumor proliferation, and induced apoptosis (Fig. S6F). Notably, neither cisplatin, maraviroc, nor their combination affected CCR5 expression (Fig. S6F).

Since maraviroc did not exhibit apparent toxic effects in mice, as indicated by stable body weight (Fig. S6G), we next examined its effect in NEPC mice. Castrated mice aged 8-week were treated with PBS, maraviroc, cisplatin, or a combination of both. While maraviroc alone had a minimal effect, the combination treatment significantly extended overall survival without altering body weight (Figs. 7I and S6H). Western blot analysis of tumor samples revealed that cisplatin moderately increased both γH2AX and cleaved caspase-3, despite some heterogeneity between individual samples (Fig. 7J). Cisplatin also induced CCL5 expression and AKT phosphorylation, which may limit its efficacy. Notably, combining maraviroc with cisplatin suppressed AKT phosphorylation, thereby enhancing DNA damage and apoptosis (Fig. 7J).

To further validate these findings, we transitioned from genetically modified mouse models to patient-derived xenografts from NEPC tumors. Similar to the allograft tumors above mentioned, cisplatin increased CCL5 expression, which largely overlapped with αSMA, suggesting that CAFs were the main source of CCL5 (Figs. 7K, S6I–L). However, the uneven growth rate of patient-derived xenograft tumors limited the direct evaluation of treatment effects. We instead generated organoids from patient-derived xenograft tumors and treated them with PBS, maraviroc, cisplatin, or their combination in vitro. As shown in Fig. 7L, cisplatin treatment inhibited organoid proliferation and reduced organoid size, as reflected by CellTiter-Glo data and representative brightfield images. Importantly, the combination of cisplatin with maraviroc significantly enhanced the inhibitory effect compared with cisplatin alone (Fig. 7L). Western blot analysis of organoids further demonstrated that maraviroc inhibited cisplatin-induced AKT phosphorylation and the combination of maraviroc and cisplatin notably increased DNA damage and apoptosis, as indicated by elevated γH2AX and cleaved caspase-3 levels, compared with cisplatin alone (Fig. 7M). Together, these findings suggest that targeting the CCL5/CCR5 axis enhances the efficacy of cisplatin treatment in NEPC, providing a strong rationale for further clinical development.

## Discussion

Chemoresistance remains a major challenge in NEPC treatment [47], highlighting the significance of understanding its mechanisms. In this study, we uncover a crosstalk between tumor cells and CAFs that enables tumors to survive cisplatin treatment. We identify a cGAS-STING–dependent mechanism driving CCL5 secretion in cisplatin-induced senescent CAFs. These senescent CAFs act as a double-edged sword: despite undergoing transient cell cycle arrest, they paradoxically produce senescence-associated secretory phenotype factors such as CCL5. Secreted CCL5 binds to CCR5 on tumor cells, activating the PI3K/AKT pathway to potential DNA repair, hence protecting tumor cells from cisplatin-induced cell death. Our findings reveal the critical role of cGAS-STING in mediating the secretion of CCL5, which facilitates intercellular communication between tumor cells and stromal cells during cisplatin treatment, although we do not exclude the possibility that other pathways may also contribute.

While CAFs’ role in chemoresistance has been reported in cancers such as ovarian and lung cancer, the molecular mechanisms are still open questions [48–51]. Our findings provide new insights into the CCL5/CCR5 paracrine axis in NEPC, identifying it as an essential mediator of stromal-tumor communication via the PI3K/AKT pathway. Although it is well established that CCL5 activates CCR5, and that CCR5 interacts with β-arrestin1, as well as β-arrestin1 with p85 [44, 46], the mechanism by which the CCL5/CCR5 axis activates the PI3K/AKT pathway and contributes to DNA damage response and anti-apoptotic activity remains unclear. We show that CCL5 remarkably activates the AKT phosphorylation in NEPC cells, and both CCR5 and β-arrestin1 are required in this progress (Figs. 5B –D, 6A and B). Exogenous and endogenous co-immunoprecipitations confirm that CCL5 stimulates the formation of the CCR5–β-arrestin1–p85, leading to AKT activation (Figs. 6F–I). Given the essential role of PI3K/AKT in cell survival, DNA repair, and resistance to apoptosis [52–55], our findings link the CCL5/CCR5 signaling to the protective effect of cisplatin-induced cell death and add a comprehensive description of CCL5-induced CCR5/β-arrestin1/PI3K/AKT cascade in chemoresistance.

Considering the crucial effect of CCL5/CCR5 on the response to cisplatin treatment, we repurpose CCR5 antagonist maraviroc to enhance the chemotherapeutic efficacy of NEPC and observe a remarkable improvement in tumor suppression in the NEPC mouse model. Maraviroc is an FDA-approved antiretroviral drug originally developed for the treatment of HIV infection [35]. Preclinical studies have highlighted the promising role of maraviroc in anti-tumor therapies through the CCL5/CCR5 pathway, including breast cancer, colon cancer, glioblastoma, and gastric cancer [56–60]. For example, maraviroc combined with cisplatin has been shown to reduce tumor cell viability, suppress spheroid growth, and improve survival in mouse models compared with cisplatin alone [60]. Beyond its direct effects on tumor cells, maraviroc also modulates the tumor immune microenvironment, repolarizing macrophages from an M2 to an M1 phenotype and alleviating myeloid-driven immunosuppression [61]. Moreover, maraviroc has been reported to interact with ATP-binding cassette (ABC) transporters such as ABCB1, which are critical mediators of drug efflux, thereby potentially prolonging intracellular cisplatin retention [62]. These diversified mechanisms suggest that maraviroc holds broad therapeutic potential in anti-tumor therapies. At the same time, they also highlight the importance of evaluating possible side effects, especially when considering drug repurposing. In our NEPC models, no changes in body weight were observed in mice treated with maraviroc alone or in combination with cisplatin (Figs. S6G and H). Nonetheless, a more comprehensive safety assessment is warranted to better define its tolerability and clinical translational potential.

While previous studies in prostate adenocarcinoma [63] have shown that CCL5 upregulates androgen receptor signaling and contributes to enzalutamide resistance—an effect that may be mitigated by maraviroc—our current study focuses on the role of the unique context of cisplatin treatment in NEPC. NEPC represents a clinically and biologically distinct subtype of prostate cancer, differing markedly from enzalutamide-resistant adenocarcinoma in histopathology, treatment strategies, prognosis, and underlying molecular drivers [9, 24, 64, 65].

However, the current research still has some limitations, including a lack of suitable human NEPC cell lines. Because the patient-derived NEPC cell lines H660 exhibit poor in vitro proliferation ratio and low lentiviral transduction efficiency, we instead employed the MYCN/AKT1-driven LASCPC-01 cell to investigate the intercellular crosstalk and underlying mechanism in NEPC. Another limitation of our study is that it focuses exclusively on the CCL5/CCR5 signaling and maraviroc’s effect in cisplatin treatment in NEPC. Given cisplatin’s widespread use as a first-line chemotherapeutic agent across various cancers, further investigation is warranted to evaluate the broader clinical potential of maraviroc in other tumor types.

## Conclusions

Our study shows that in NEPC, CAF-derived CCL5 binds to CCR5 on tumor cells, activating the PI3K/AKT pathway to protect cells from DNA damage and cisplatin-induced cell death. Maraviroc disrupts CCL5/CCR5 signaling and blocks the protective effect, enhancing the efficacy of cisplatin-based chemotherapy.

## Supplementary Information

Additional file 1.  Supplemental figures and tables

Additional file 2.  Source data

## Data Availability

The bulk RNA sequencing data generated in this study have been uploaded to the Gene Expression Omnibus database under accession number GSE287963. All other raw data are available upon request from the corresponding author.

## Abbreviations

NEPC: Neuroendocrine prostate cancer
CAFs: Cancer-associated fibroblasts
αSMA: Alpha smooth muscle actin
EpCAM: Epithelial cell adhesion molecule
PDX: Patient-derived xenograft
PDO: Patient-derived organoid
