# Supplementary material for: Targeting the CCL5/CCR5 axis in tumor-stromal crosstalk to overcome cisplatin resistance in neuroendocrine prostate cancer
Source: J Exp Clin Cancer Res. 2025 Oct 28;44:296. doi: 10.1186/s13046-025-03552-y (PMC12570517; doi:10.1186/s13046-025-03552-y)
Supplement: Supplementary file 1 — Supplemental figures and tables [file 13046_2025_3552_MOESM1_ESM.pdf]

Fig. S1

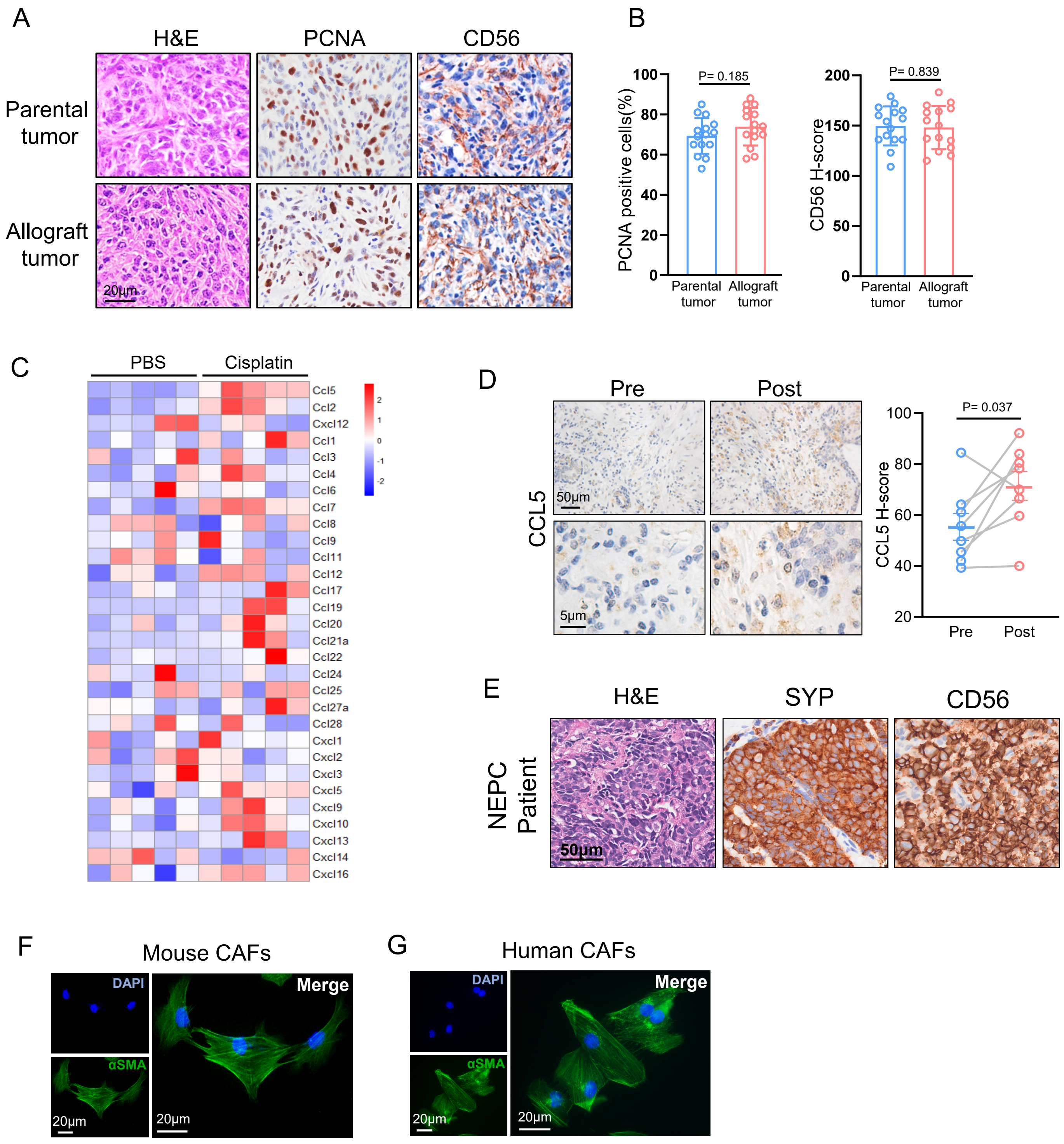
A
H&E
PCNA
CD56
Parental tumor
Allograft tumor
20μm
B
P= 0.185
PCNA positive cells(%)
100
80
60
40
20
0
Parental tumor
Allograft tumor
P= 0.839
CD56 H-score
200
150
100
50
0
Parental tumor
Allograft tumor
C
PBS
Cisplatin
Ccl5
Ccl2
Cxcl12
Ccl1
Ccl3
Ccl4
Ccl6
Ccl7
Ccl8
Ccl9
Ccl11
Ccl12
Ccl17
Ccl19
Ccl20
Ccl21a
Ccl22
Ccl24
Ccl25
Ccl27a
Ccl28
Cxcl1
Cxcl2
Cxcl3
Cxcl5
Cxcl9
Cxcl10
Cxcl13
Cxcl14
Cxcl16
2
1
0
-1
-2
D
Pre
Post
CCL5
50μm
5μm
P= 0.037
CCL5 H-score
100
80
60
40
20
Pre
Post
E
H&E
SYP
CD56
NEPC Patient
50μm
F
Mouse CAFs
DAPI
αSMA
Merge
20μm
20μm
G
Human CAFs
DAPI
αSMA
Merge
20μm
20μm

Fig. S1. Cisplatin increases CCL5 expression in cancer-associated fibroblasts

(A) Representative H&E and immunohistochemistry images of PCNA and CD56 in allograft tumors versus parental tumors (Pb-Cre4: *Pten*$^{f/f}$; *Trp53*$^{f/f}$; *Rb1*$^{f/f}$). Scale bar, 20μm.

(B) Quantification of immunohistochemistry of PCNA (left) and CD56 (right) in tumors from (A), (n = 15 per group).

(C) Heatmap showing the expression of the chemokine genes in allograft tumors treated with cisplatin or PBS.

(D) Representative immunohistochemistry images (left) and quantification (right) of CCL5 between paired pre-and post-treatment samples from patients with prostate adenocarcinoma who received neoadjuvant cisplatin-based combination therapy (n = 8 per group). Scale bars, above, 50μm; below, 5μm.

(E) Representative H&E and immunohistochemistry images of SYP and CD56 in samples from NEPC patients.

(F–G) Representative immunofluorescence images of αSMA in mouse CAFs isolated from Pb-Cre4: *Pten*$^{f/f}$; *Trp53*$^{f/f}$; *Rb1*$^{f/f}$ tumors (F) and human CAFs isolated from NEPC patient tumors (G) (blue, DAPI; green, αSMA). Scale bar, 20μm.

Data presented as mean and error bars report standard deviation (B and D). Statistical significance was determined by two-tailed unpaired Student's *t*-test (B) or two-tailed paired Student's *t*-test (D).

Fig. S2

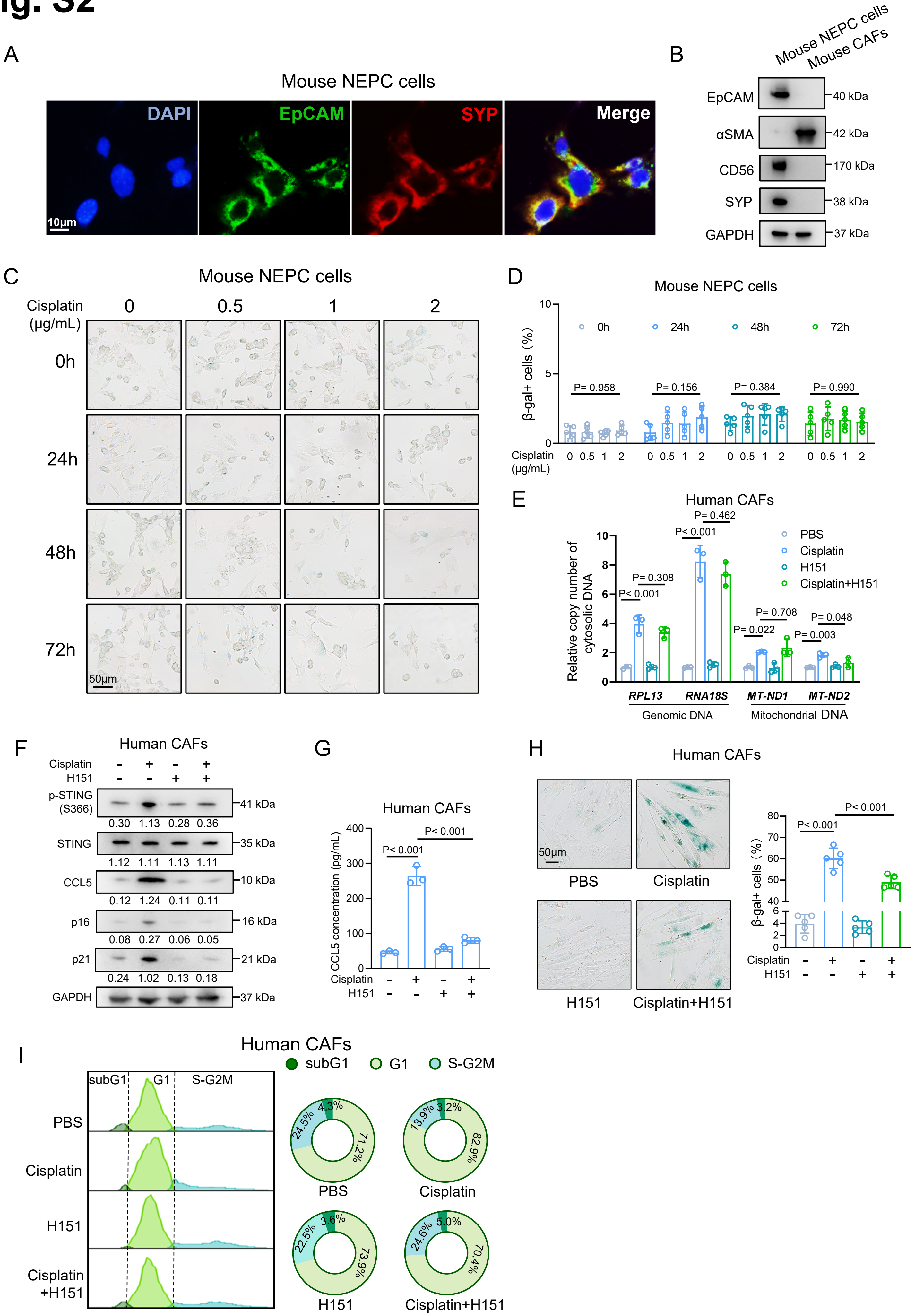
A
Mouse NEPC cells
DAPI
EpCAM
SYP
Merge
10μm
B
Mouse NEPC cells
Mouse CAFs
EpCAM
αSMA
CD56
SYP
GAPDH
40 kDa
42 kDa
170 kDa
38 kDa
37 kDa
C
Mouse NEPC cells
Cisplatin (μg/mL)
0
0.5
1
2
0h
24h
48h
72h
50μm
D
Mouse NEPC cells
β-gal+ cells (%)
0h
24h
48h
72h
P= 0.958
P= 0.156
P= 0.384
P= 0.990
Cisplatin (μg/mL)
E
Human CAFs
Relative copy number of cytosolic DNA
PBS
Cisplatin
H151
Cisplatin+H151
P= 0.462
P< 0.001
P= 0.308
P< 0.001
P= 0.708
P= 0.022
P= 0.048
P= 0.003
RPL13
RNA18S
MT-ND1
MT-ND2
Genomic DNA
Mitochondrial DNA
F
Human CAFs
Cisplatin
H151
p-STING (S366)
41 kDa
0.30 1.13 0.28 0.36
STING
35 kDa
1.12 1.11 1.13 1.11
CCL5
10 kDa
0.12 1.24 0.11 0.11
p16
16 kDa
0.08 0.27 0.06 0.05
p21
21 kDa
0.24 1.02 0.13 0.18
GAPDH
37 kDa
G
Human CAFs
CCL5 concentration (pg/mL)
P< 0.001
P< 0.001
Cisplatin
H151
H
Human CAFs
50μm
PBS
Cisplatin
H151
Cisplatin+H151
β-gal+ cells (%)
P< 0.001
P< 0.001
Cisplatin
H151
I
Human CAFs
subG1
G1
S-G2M
PBS
Cisplatin
H151
Cisplatin +H151
4.3%
24.5%
71.2%
3.2%
13.9%
82.9%
3.6%
22.5%
73.9%
5.0%
24.6%
70.4%
PBS
Cisplatin
H151
Cisplatin+H151

Fig. S2. Cisplatin predisposes CAFs to senescence via the cGAS-STING pathway

(A) Representative immunofluorescence of EpCAM and synaptophysin (SYP) in epithelial cells isolated from tumor tissues of NEPC mice (Pb-Cre4: *Pten*$^{f/f}$; *Trp53*$^{f/f}$; *Rb1*$^{f/f}$) (blue, DAPI; green, EpCAM; red, SYP). Scale bar, 10μm.

(B) Western blot showing protein expression of EpCAM, αSMA, CD56, and SYP in mouse NEPC cells and mouse CAFs.

(C) Representative β-gal staining images of mouse NEPC cells treated with cisplatin in indicated concentrations and times. Scale bar, 50μm.

(D) Quantification of the percentage of β-gal–positive mouse NEPC cells treated with cisplatin in indicated concentrations and times (n = 5 per group).

(E) QPCR analysis of genomic DNA (*RPL13* and *RNA18S*) and mitochondrial DNA (*MT-ND1* and *MT-ND2*) in human CAFs treated with PBS, cisplatin (2μg/mL), H151 (1μM), or cisplatin plus H151.

(F) Western blot showing expression of indicated proteins in human CAFs treated with PBS, cisplatin (2μg/mL), H151 (1μM), or cisplatin plus H151.

(G) ELISA assay showing the concentration of CCL5 in the supernatant of human CAFs treated with PBS, cisplatin (2μg/mL), H151 (1μM), or cisplatin plus H151.

(H) Representative images (left) and quantification (right) of β-gal staining for human CAFs with indicated treatments (n = 5 per group). Scale bar, 50μm.

(I) Cell cycle distribution by flow cytometry of human CAFs treated with PBS, cisplatin (2μg/mL), H151 (1μM), or cisplatin plus H151.

Data presented as mean and error bars reported standard deviation (D, E, G, and H). Statistical significance was determined by ANOVA with Dunnett's multiple comparisons (D, E, G, and H). The protein levels were normalized to the GAPDH using ImageJ. Western blot experiments were repeated three times independently, with similar results (B and F).

Fig. S3

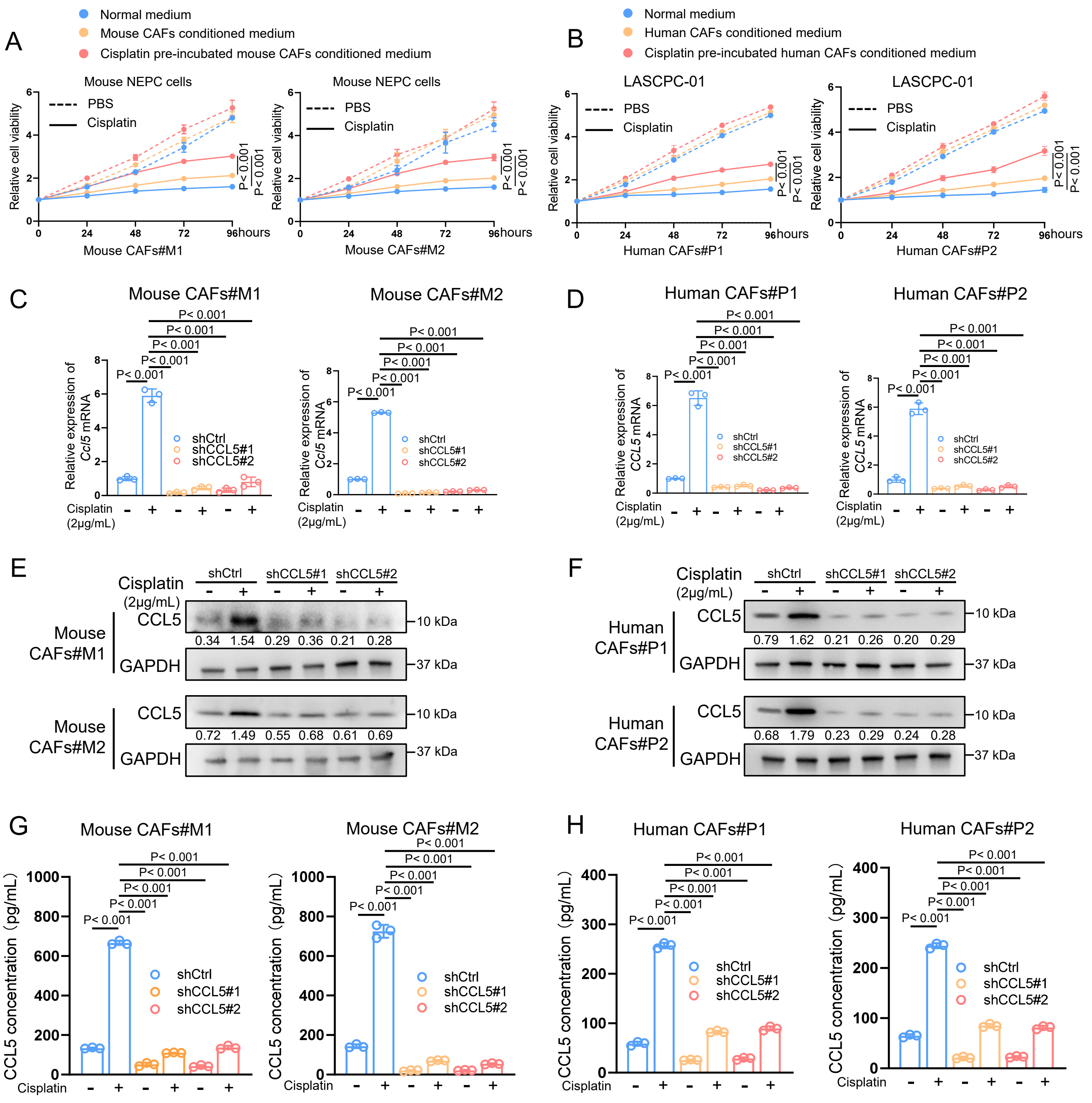

A
Normal medium
Mouse CAFs conditioned medium
Cisplatin pre-incubated mouse CAFs conditioned medium
Mouse NEPC cells
PBS
Cisplatin
Relative cell viability
P< 0.001
Mouse CAFs#M1
Mouse CAFs#M2
hours
B
Normal medium
Human CAFs conditioned medium
Cisplatin pre-incubated human CAFs conditioned medium
LASCPC-01
Human CAFs#P1
Human CAFs#P2
C
Mouse CAFs#M1
Mouse CAFs#M2
Relative expression of Ccl5 mRNA
shCtrl
shCCL5#1
shCCL5#2
Cisplatin (2μg/mL)
D
Human CAFs#P1
Human CAFs#P2
Relative expression of CCL5 mRNA
E
Cisplatin (2μg/mL)
shCtrl
shCCL5#1
shCCL5#2
Mouse CAFs#M1
CCL5
0.34 1.54 0.29 0.36 0.21 0.28
GAPDH
10 kDa
37 kDa
Mouse CAFs#M2
0.72 1.49 0.55 0.68 0.61 0.69
F
Human CAFs#P1
0.79 1.62 0.21 0.26 0.20 0.29
Human CAFs#P2
0.68 1.79 0.23 0.29 0.24 0.28
G
Mouse CAFs#M1
Mouse CAFs#M2
CCL5 concentration (pg/mL)
Cisplatin
H
Human CAFs#P1
Human CAFs#P2

Fig. S3. CAFs-derived CCL5 mediates chemotherapy resistance of cancer cells

(A) Cell viability of mouse NEPC cells cultured with indicated medium and treated with cisplatin or PBS control. Conditioned medium was collected from mouse CAFs pre-incubated with cisplatin or PBS.

(B) Cell viability of LASCPC-01 cultured with indicated medium and treated with cisplatin or PBS control. Conditioned medium was collected from human CAFs pre-incubated with cisplatin or PBS.

(C) QPCR showing relative mRNA expression of CCL5 in mouse CAFs with control shRNA or two independent shRNAs targeting CCL5, followed by cisplatin or PBS treatment.

(D) QPCR showing relative mRNA expression of CCL5 in human CAFs with control shRNA or two independent shRNAs targeting CCL5, followed by cisplatin or PBS treatment.

(E) Western blot showing protein expression of CCL5 in mouse CAFs with control shRNA or two independent shRNAs targeting CCL5, followed by cisplatin or PBS treatment.

(F) Western blot showing protein expression of CCL5 in human CAFs with control shRNA or two independent shRNAs targeting CCL5, followed by cisplatin or PBS treatment.

(G) ELISA assay showing the concentration of CCL5 in the supernatant of mouse CAFs with control shRNA or two independent shRNAs targeting CCL5, followed by cisplatin or PBS treatment.

(H) ELISA assay showing the concentration of CCL5 in the supernatant of human CAFs with control shRNA or two independent shRNAs targeting CCL5, followed by cisplatin or PBS treatment.

Data presented as mean and error bars reported standard deviation (A–D, and G–H). Statistical significance was determined by one-way analysis of variance (ANOVA) with Dunnett’s multiple comparisons (A–D, and G–H). CAFs#M1 and CAFs#M2 are mouse CAF strains isolated from two NEPC mice (Pb-Cre4: *Pten*$^{f/f}$; *Trp53*$^{f/f}$; *Rb1*$^{f/f}$) (A, C, E, and G). CAFs#P1 and CAFs#P2 are human CAF strains isolated from two NEPC patients (B, D, F, and H). Two independent shRNA sequences targeting CCL5 mRNA were labeled as shCCL5#1 and shCCL5#2 (E–H). The protein levels were normalized to the GAPDH using ImageJ. Western blot experiments were repeated three times independently, with similar results (E and F).

Fig. S4

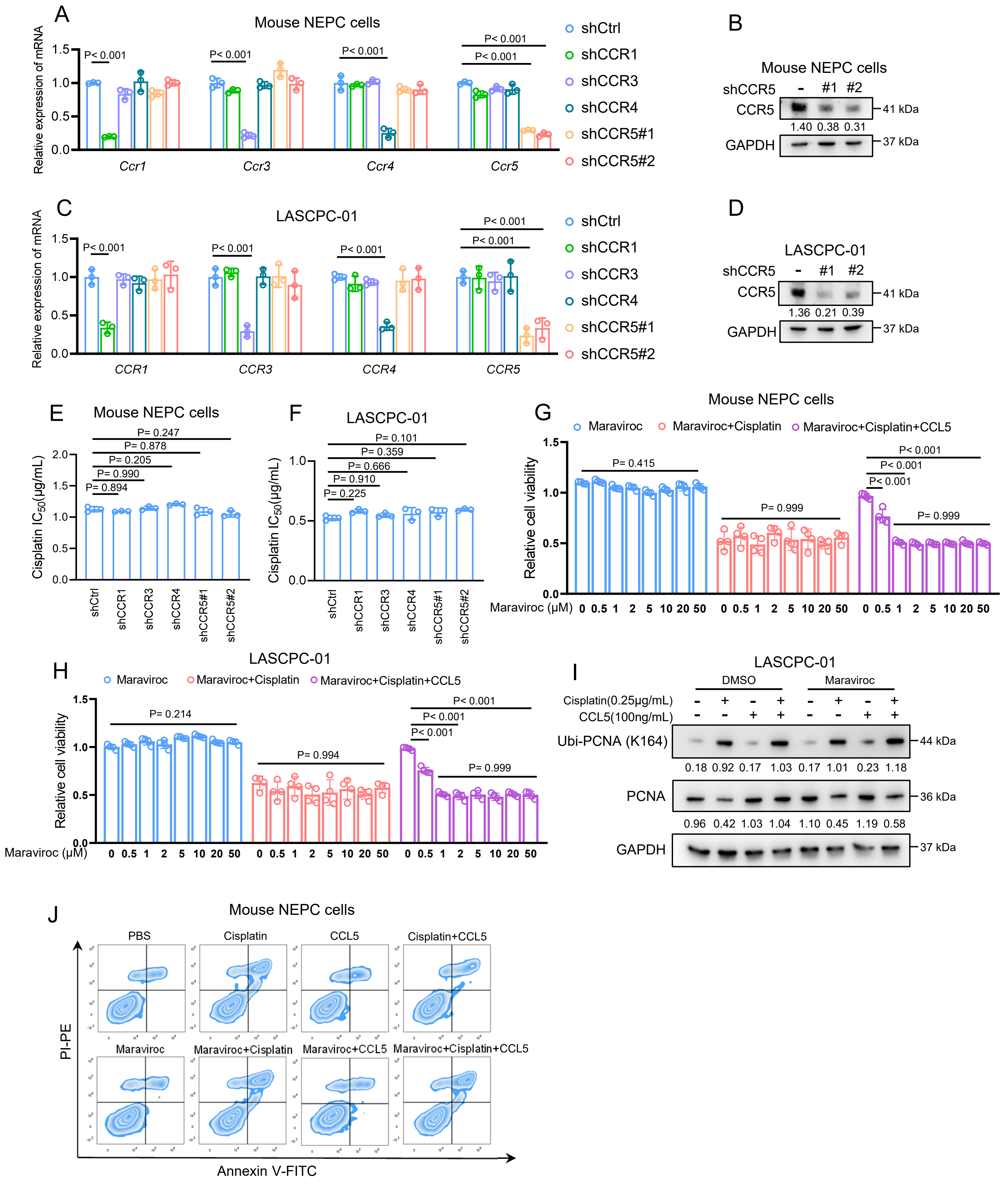

A
Mouse NEPC cells
Relative expression of mRNA
Ccr1
Ccr3
Ccr4
Ccr5
P< 0.001
shCtrl
shCCR1
shCCR3
shCCR4
shCCR5#1
shCCR5#2
B
Mouse NEPC cells
shCCR5 - #1 #2
CCR5
1.40 0.38 0.31
GAPDH
41 kDa
37 kDa
C
LASCPC-01
CCR1
CCR3
CCR4
CCR5
D
LASCPC-01
1.36 0.21 0.39
E
Mouse NEPC cells
Cisplatin IC50(μg/mL)
P= 0.247
P= 0.878
P= 0.205
P= 0.990
P= 0.894
F
LASCPC-01
P= 0.101
P= 0.359
P= 0.666
P= 0.910
P= 0.225
G
Mouse NEPC cells
Maraviroc
Maraviroc+Cisplatin
Maraviroc+Cisplatin+CCL5
Relative cell viability
P= 0.415
P= 0.999
P< 0.001
Maraviroc (μM) 0 0.5 1 2 5 10 20 50
H
LASCPC-01
P= 0.214
P= 0.994
I
LASCPC-01
DMSO
Maraviroc
Cisplatin(0.25μg/mL)
CCL5(100ng/mL)
Ubi-PCNA (K164)
0.18 0.92 0.17 1.03 0.17 1.01 0.23 1.18
PCNA
0.96 0.42 1.03 1.04 1.10 0.45 1.19 0.58
GAPDH
44 kDa
36 kDa
37 kDa
J
Mouse NEPC cells
PBS
Cisplatin
CCL5
Cisplatin+CCL5
Maraviroc
Maraviroc+Cisplatin
Maraviroc+CCL5
Maraviroc+Cisplatin+CCL5
PI-PE
Annexin V-FITC

Fig. S4. The CCL5-CCR5 axis protects cancer cells against cisplatin-induced DNA damage and apoptosis

(A) QPCR showing relative mRNA expression of CCR1, CCR3, CCR4, and CCR5 in indicated mouse NEPC cells.

(B) Western blot showing protein expression of CCR5 in indicated mouse NEPC cells.

(C) QPCR showing relative mRNA expression of CCR1, CCR3, CCR4, and CCR5 in indicated LASCPC-01 cells.

(D) Western blot showing protein expression of CCR5 in indicated LASCPC-01.

(E) IC_{50} values of cisplatin in indicated mouse NEPC cells.

(F) IC_{50} values of cisplatin in indicated LASCPC-01 cells.

(G–H) Cell viability of mouse NEPC cells (G) and LASCPC-01 (H) with indicated treatments for 48 hours.

(I) Western blot showing ubiquitination and global expression of PCNA in LASCPC-01 with indicated treatments.

(J) Representative flow cytometry plots of Annexin V/propidium iodide staining in mouse NEPC cells with indicated treatments shown in Fig. 4Q.

Data presented as mean and error bars reported standard deviation (A, C, and E–H). Statistical significance was determined by ANOVA with Dunnett's multiple comparisons (A, C, and E–H). Two independent shRNA sequences targeting CCR5 mRNA were labeled as shCCR5#1 and shCCR5#2 (A–F). The protein levels were normalized to the GAPDH using ImageJ. Western blot experiments were repeated three times independently, with similar results (B, D and I).

Fig. S5

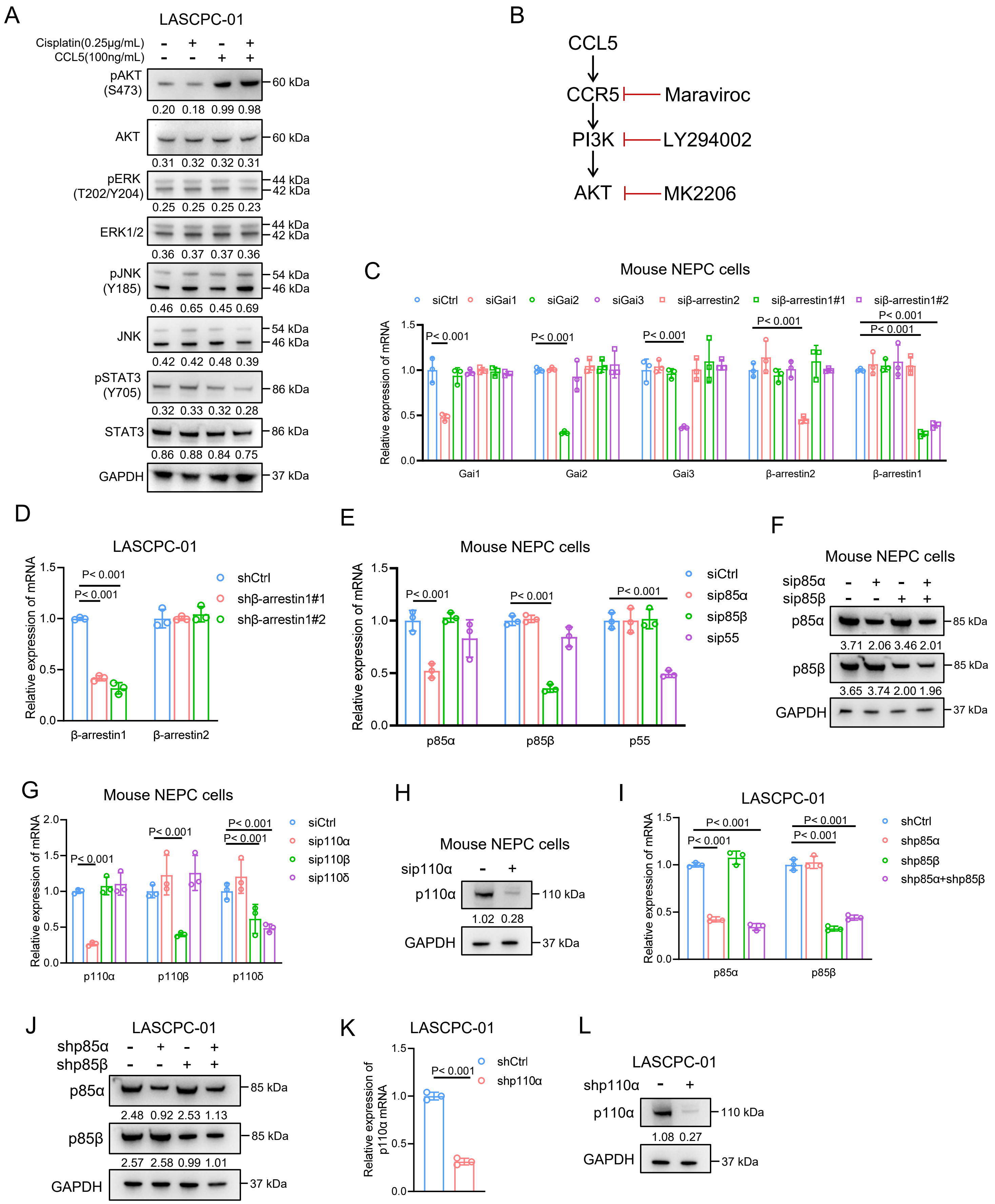
A
LASCPC-01
Cisplatin(0.25μg/mL) - + - +
CCL5(100ng/mL) - - + +
pAKT (S473) 60 kDa
0.20 0.18 0.99 0.98
AKT 60 kDa
0.31 0.32 0.32 0.31
pERK (T202/Y204) 44 kDa 42 kDa
0.25 0.25 0.25 0.23
ERK1/2 44 kDa 42 kDa
0.36 0.37 0.37 0.36
pJNK (Y185) 54 kDa 46 kDa
0.46 0.65 0.45 0.69
JNK 54 kDa 46 kDa
0.42 0.42 0.48 0.39
pSTAT3 (Y705) 86 kDa
0.32 0.33 0.32 0.28
STAT3 86 kDa
0.86 0.88 0.84 0.75
GAPDH 37 kDa
B
CCL5
CCR5 Maraviroc
PI3K LY294002
AKT MK2206
C
Mouse NEPC cells
siCtrl siGai1 siGai2 siGai3 siβ-arrestin2 siβ-arrestin1#1 siβ-arrestin1#2
Relative expression of mRNA
P< 0.001
Gai1 Gai2 Gai3 β-arrestin2 β-arrestin1
D
LASCPC-01
shCtrl shβ-arrestin1#1 shβ-arrestin1#2
Relative expression of mRNA
P< 0.001
β-arrestin1 β-arrestin2
E
Mouse NEPC cells
siCtrl sip85α sip85β sip55
Relative expression of mRNA
P< 0.001
p85α p85β p55
F
Mouse NEPC cells
sip85α - + - +
sip85β - - + +
p85α 85 kDa
3.71 2.06 3.46 2.01
p85β 85 kDa
3.65 3.74 2.00 1.96
GAPDH 37 kDa
G
Mouse NEPC cells
siCtrl sip110α sip110β sip110δ
Relative expression of mRNA
P< 0.001
p110α p110β p110δ
H
Mouse NEPC cells
sip110α - +
p110α 110 kDa
1.02 0.28
GAPDH 37 kDa
I
LASCPC-01
shCtrl shp85α shp85β shp85α+shp85β
Relative expression of mRNA
P< 0.001
p85α p85β
J
LASCPC-01
shp85α - + - +
shp85β - - + +
p85α 85 kDa
2.48 0.92 2.53 1.13
p85β 85 kDa
2.57 2.58 0.99 1.01
GAPDH 37 kDa
K
LASCPC-01
shCtrl shp110α
Relative expression of p110α mRNA
P< 0.001
L
LASCPC-01
shp110α - +
p110α 110 kDa
1.08 0.27
GAPDH 37 kDa

Fig. S5. CCL5 induces AKT phosphorylation by promoting the formation of CCR5/β-arrestin1/p85 complex

(A) Western blot showing protein expression of phosphorylated AKT(S473), AKT, phosphorylated ERK(T202/Y204), ERK, phosphorylated JNK(Y185), JNK, phosphorylated STAT3 (Y705), and STAT3 in LASCPC-01 treated with PBS, CCL5(100ng/mL), cisplatin (0.25μg/mL), or CCL5 plus cisplatin.

(B) Schematic showing the experimental strategy used to confirm the CCR5/PI3K/AKT transduction cascade.

(C) QPCR showing relative mRNA expression of Gai1, Gai2, Gai3, β-arrestin1, and β-arrestin2 in indicated mouse NEPC cells.

(D) QPCR showing relative mRNA expression of β-arrestin1 and β-arrestin2 in indicated LASCPC-01.

(E) QPCR showing relative mRNA expression of p85α, p85β, and p55 in indicated mouse NEPC cells.

(F) Western blot showing protein expression of p85α and p85β in indicated mouse NEPC cells.

(G) QPCR showing relative mRNA expression of p110α, p110β, and p110δ in indicated mouse NEPC cells.

(H) Western blot showing protein expression of p110α in indicated mouse NEPC cells.

(I) QPCR showing relative mRNA expression of p85α and p85β in indicated LASCPC-01.

(J) Western blot showing protein expression of p85α and p85β in indicated LASCPC-01.

(K) QPCR showing relative mRNA expression of p110α in indicated LASCPC-01.

(L) Western blot showing protein expression of p110α in indicated LASCPC-01.

Data presented as mean and error bars reported standard deviation (C–E, G, I, and K). Statistical significance was determined by ANOVA with Dunnett's multiple comparisons (C–E, G, and I) or two-tailed unpaired Student's *t*-test (K). Two independent siRNA sequences targeting β-arrestin1 mRNA were labeled as siβ-arrestin1#1 and siβ-arrestin1#2 (C). Two independent shRNA sequences targeting β-arrestin1 mRNA were labeled as shβ-arrestin1#1 and shβ-arrestin1#2 (D). The protein levels were normalized to the GAPDH using ImageJ. Western blot experiments were repeated three times independently, with similar results (A, F, H, J, and L).

Fig. S6

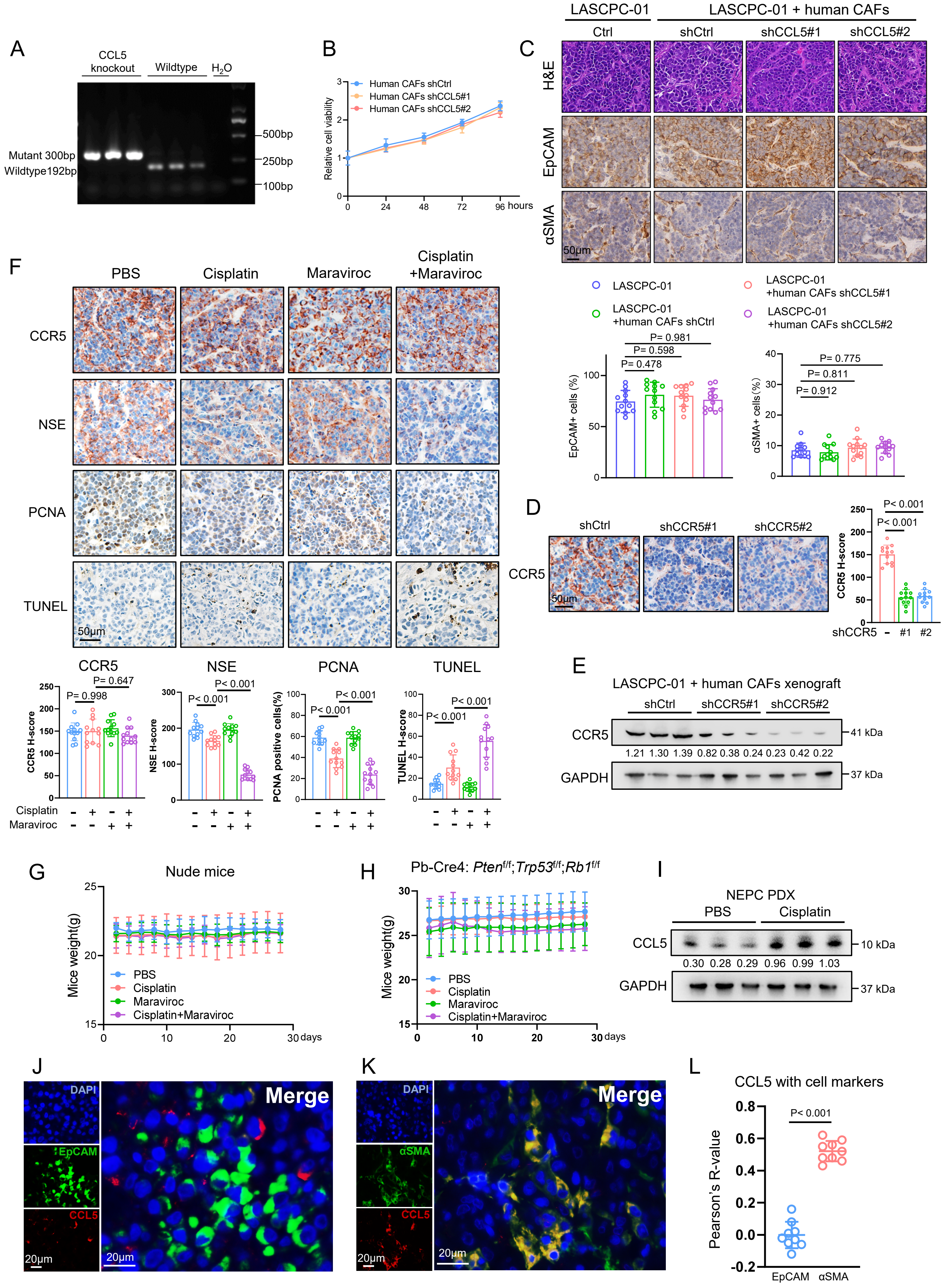

A
CCL5 knockout
Wildtype
H2O
Mutant 300bp
Wildtype192bp
500bp
250bp
100bp
B
Human CAFs shCtrl
Human CAFs shCCL5#1
Human CAFs shCCL5#2
Relative cell viability
hours
C
LASCPC-01
LASCPC-01 + human CAFs
Ctrl
shCtrl
shCCL5#1
shCCL5#2
H&E
EpCAM
αSMA
50μm
LASCPC-01
LASCPC-01 +human CAFs shCtrl
LASCPC-01 +human CAFs shCCL5#1
LASCPC-01 +human CAFs shCCL5#2
P= 0.981
P= 0.598
P= 0.478
EpCAM+ cells (%)
P= 0.775
P= 0.811
P= 0.912
αSMA+ cells (%)
D
shCtrl
shCCR5#1
shCCR5#2
CCR5
50μm
P< 0.001
P< 0.001
CCR5 H-score
shCCR5
#1
#2
E
LASCPC-01 + human CAFs xenograft
shCtrl
shCCR5#1
shCCR5#2
CCR5
1.21 1.30 1.39 0.82 0.38 0.24 0.23 0.42 0.22
GAPDH
41 kDa
37 kDa
F
PBS
Cisplatin
Maraviroc
Cisplatin +Maraviroc
CCR5
NSE
PCNA
TUNEL
50μm
CCR5
P= 0.647
P= 0.998
CCR5 H-score
NSE
P< 0.001
P< 0.001
NSE H-score
PCNA
P< 0.001
P< 0.001
PCNA positive cells(%)
TUNEL
P< 0.001
P< 0.001
TUNEL H-score
Cisplatin
Maraviroc
G
Nude mice
Mice weight(g)
PBS
Cisplatin
Maraviroc
Cisplatin+Maraviroc
days
H
Pb-Cre4: Pten f/f;Trp53 f/f;Rb1 f/f
Mice weight(g)
PBS
Cisplatin
Maraviroc
Cisplatin+Maraviroc
days
I
NEPC PDX
PBS
Cisplatin
CCL5
0.30 0.28 0.29 0.96 0.99 1.03
GAPDH
10 kDa
37 kDa
J
DAPI
EpCAM
CCL5
Merge
20μm
K
DAPI
αSMA
CCL5
Merge
20μm
L
CCL5 with cell markers
P< 0.001
Pearson's R-value
EpCAM
αSMA

Fig. S6. Targeting the CCL5/CCR5 pathway overcomes cisplatin resistance and enhances chemotherapy efficiency

(A) PCR-based genotyping assay of tissues from CCL5 knockout or wildtype mice tails.

(B) Cell viability of human CAFs with shCtrl, shCCL5#1, and shCCL5#2.

(C) Representative immunohistochemistry images (above) and quantification (below) of EpCAM positive cells and αSMA positive cells in LASCPC-01 xenografts (n = 12 fields from 3 mice per treatment condition) in Fig. 7F. Scale bar, 50μm.

(D) Representative immunohistochemistry images (left) and quantification (right) of CCR5 in LASCPC-01/human CAFs co-injection tumor model. Scale bar, 50μm.

(E) Western blot showing protein expression of CCR5 in tumors in LASCPC-01/human CAFs co-injection tumor model.

(F) Representative immunohistochemistry images (above) and corresponding quantification (below) of CCR5, NSE, PCNA, and TUNEL in LASCPC-01/human CAFs co-injection tumor model shown in Fig. 7H, under the indicated treatments (n = 12 fields from 3 mice per treatment condition). Scale bar, 50μm.

(G) Body weight changes of nude mice with indicated treatment in Fig. 7H.

(H) Body weight changes of NEPC mice (Pb-Cre4: $Pten^{f/f}$; $Trp53^{f/f}$; $Rb1^{f/f}$) in Fig. 7I, treated with PBS (n = 11), cisplatin (n = 10), maraviroc (n = 10), or cisplatin plus maraviroc (n = 10).

(I) Western blot showing protein expression of CCL5 in NEPC PDX tumors treated with cisplatin or PBS.

(J–K) Representative immunofluorescence images of CCL5 co-staining with EpCAM (J) or αSMA (K) in NEPC PDX tumors treated with cisplatin (blue, DAPI; green, EpCAM/αSMA; red, CCL5).

(L) Quantification of CCL5 co-staining with EpCAM or αSMA. The correlation was expressed by Pearson's R-value (n = 9 per group).

Data presented as mean and error bars reported standard deviation (B–D, F, G, H, and L). Statistical significance was determined by ANOVA with Dunnett's multiple comparisons (B–D and F) or two-tailed unpaired Student's *t*-test (L). Two independent shRNA sequences targeting CCL5 mRNA were labeled as shCCL5#1 and shCCL5#2 (B). Two independent shRNA sequences targeting CCR5 mRNA were labeled as shCCR5#1 and shCCR5#2 (D and E). The protein levels were normalized to the GAPDH using ImageJ. Western blot experiments were repeated three times independently, with similar results (E and I).

Table S1 Patient information.

Patient ID	Application	Collection Type	Age	Histology	Treatment
1	PDX/PDO#1;CAFs#P1	biopsy	50	small cell NEPC	Naive
2	PDO#2	biopsy	90	small cell NEPC	Naive
3	CAFs#P2	biopsy	84	CRPC-NED	ADT

CRPC: castration-resistant prostate cancer; NED: neuroendocrine differentiation; ADT: androgen deprivation therapy

Table S2 Antibodies used in this study.

Primary antibodies name	Source	Catalog	Host	Dilution	Experiment
CCL5	R&D	AF478;AF-278-NA	Goat	1:500	Western blot
CD56	proteintech	14255-1-AP	Rabbit	1:1000	Western blot
p-STING	CST	72971;19781	Rabbit	1:1000	Western blot
STING	CST	13647	Rabbit	1:1000	Western blot
GAPDH	proteintech	10494-1-AP	Rabbit	1:5000	Western blot
p16	Abcam	ab108349	Rabbit	1:1000	Western blot
p21	CST	2947	Rabbit	1:1000	Western blot
γH2AX	CST	2577	Rabbit	1:1000	Western blot
p-AKT(S473)	CST	4060	Rabbit	1:1000	Western blot
p-AKT(T308)	CST	13038	Rabbit	1:1000	Western blot
AKT	CST	4691	Rabbit	1:1000	Western blot
Cleaved caspase-3	CST	9661	Rabbit	1:1000	Western blot
HA	proteintech	51064-2-AP	Rabbit	1:1000	Western blot
V5	CST	13202	Rabbit	1:1000	Western blot
Flag	proteintech	20543-1-AP	Rabbit	1:8000	Western blot
p110α	proteintech	67071-1-Ig	Mouse	1:1000	Western blot
CCR5	proteintech	17476-1-AP	Rabbit	1:1000	Western blot
β-arrestin1	CST	4674	Rabbit	1:1000	Western blot
p85	CST	4292	Rabbit	1:1000	Western blot
p85α	proteintech	60225-1-Ig	Mouse	1:1000	Western blot
p85β	proteintech	67644-1-Ig	Mouse	1:1000	Western blot
αSMA	Abcam	ab179467	Rabbit	1:5000	Western blot
EpCAM	Abcam	ab71916	Rabbit	1:5000	Western blot
PCNA	proteintech	10205-2-AP	Rabbit	1:2000	Western blot
Ubiquityl-PCNA (K164)	CST	13439	Rabbit	1:1000	Western blot
ERK1/2	proteintech	11257-1-AP	Rabbit	1:1000	Western blot
p-ERK(T202/Y204)	proteintech	28733-1-AP	Rabbit	1:1000	Western blot
JNK	proteintech	66210-1-Ig	Mouse	1:1000	Western blot
p-JNK(Y185)	proteintech	80024-1-RR	Rabbit	1:1000	Western blot
STAT3	abclonal	A19566	Rabbit	1:1000	Western blot
p-STAT3(Y705)	CST	9145	Rabbit	1:1000	Western blot
Flag	sigma	F3165	Mouse	1:400	Immunoprecipitation
p85	proteintech	60225-1-Ig	Mouse	1:100	Immunoprecipitation
CCL5	R&D	AF478;AF-278-NA	Goat	1:50	Immunohistochemistry
EpCAM	Abcam	ab71916	Rabbit	1:2000	Immunohistochemistry
αSMA	Abcam	ab7817	Rabbit	1:2000	Immunohistochemistry
PCNA	proteintech	10205-2-AP	Rabbit	1:1000	Immunohistochemistry
EpCAM	Abcam	ab71916	Rabbit	1:3000	Immunofluoresence
CD45	proteintech	20103-1-AP	Rabbit	1:100	Immunofluoresence
αSMA	Abcam	ab7817	Rabbit	1:3000	Immunofluoresence
SYP	Abcam	ab32127	Rabbit	1:100	Immunofluoresence
γH2AX	CST	2577	Rabbit	1:200	Immunofluoresence
p-AKT(S473)	CST	4060	Rabbit	1:100	Immunofluoresence
RAD51	proteintech	67024-1-Ig	Mouse	1:100	Immunofluoresence
Pericentrin	proteintech	22271-1-AP	Rabbit	1:50	Immunofluoresence
α-tubulin	proteintech	66031-1-Ig	Mouse	1:200	Immunofluoresence
**Secondary antibodies name**	**Source**	**Catalog**	**Host**	**Dilution**	**Experiment**
Donkey Anti-Goat	Beyotime	A0181	Donkey	1:500	Western blot
Donkey Anti-Goat	Beyotime	A0181	Donkey	1:200	Immunohistochemistry
Goat Anti-Mouse	Jackson	115-035-003	Goat	1:5000	Western blot
Goat Anti-Rabbit	Jackson	111-035-003	Goat	1:5000	Western blot
Goat Anti-Rabbit	Jackson	111-035-003	Goat	1:500	Immunohistochemistry
Goat Anti-Rabbit	Abcam	ab150077	Goat	1:500	Immunofluoresence
Goat Anti-Rabbit	Abcam	ab150080	Goat	1:500	Immunofluoresence
Donkey Anti-Mouse AF488	Invitrogen	A-21202	Donkey	1:500	Immunofluoresence
Donkey Anti-Rabbit AF594	Invitrogen	A-21207	Donkey	1:500	Immunofluoresence

Table S3 Primers and oligos used in this study.

QPCR primers		
**Name**	**Species**	**Sequence 5' to 3'**
CCL5(F)	human	CCTGCTGCTTTGCCTACATTGC
CCL5(R)	human	ACACACTTGGCGGTTCTTTCGG
Ccl5(F)	mouse	CCTGCTGCTTTGCCTACCTCTC
Ccl5(R)	mouse	ACACACTTGGCGGTTCCTTCGA
CCR1(F)	human	CAACTCCGTGCCAGAAGGTGAA
CCR1(R)	human	GTTCAGGAGGTAGATGCTGGTC
Ccr1(F)	mouse	GCCAAAAGACTGCTGTAAGAGCC
Ccr1(R)	mouse	GCTTTGAAGCCTCCTATGCTGC
CCR3(F)	human	TACTCCCTGGTGTTCACTGTGG
CCR3(R)	human	ACGAGGAAGAGCAGGTCCGAAA
Ccr3(F)	mouse	CCACTGTACTCCCTGGTGTTCA
Ccr3(R)	mouse	GGACAGTGAAGAGAAAGAGCAGG
CCR4(F)	human	CTCTGGCTTTTGTTCACTGCTGC
CCR4(R)	human	AGCCCACAGTATTGGCAGAGCA
Ccr4(F)	mouse	GGACTAGGTCTGTGCAAGATCG
Ccr4(R)	mouse	TGCCTTCAAGGAGAATACCGCG
CCR5(F)	human	TCTCTTCTGGGCTCCCTACAAC
CCR5(R)	human	CCAAGAGTCTCTGTCACCTGCA
Ccr5(F)	mouse	GTCTACTTTCTCTTCTGGACTCC
Ccr5(R)	mouse	CCAAGAGTCTCTGTTGCCTGCA
RPL13(F)	human	GTAACCCGTTGAACCCCATT
RPL13(R)	human	GCGATGATGGCTAACCTACC
Rpl13(F)	mouse	GTGGACACTTGGTTCAACCAGC
Rpl13(R)	mouse	GGTGTGGTATCTCACTGTAGGG
RNA18S(F)	human	ACCCGTTGAACCCCATTCGTGA
RNA18S(R)	human	GCCTCACTAAACCATCCAATCGG
Rn18s(F)	mouse	TTGGCTGGTTTGGGAGGTTT
Rn18s(R)	mouse	ACTGGCCGACCTCCTTATCT
Mt-nd1(F)	mouse	TAACCGCATCGGAGACATCG
Mt-nd1(R)	mouse	AGGACTGGAATGCTGGTTGG
MT-ND1(F)	human	CTCTTCGTCTGATCCGTCCT
MT-ND1(R)	human	TGATTGTCTGGCGTTGGAGT
Mt-nd2(F)	mouse	GCTAGCCGCAGGCATTACTA
Mt-nd2(R)	mouse	CTCCGTGTAGGGTTGCAAGT
MT-ND2(F)	human	GTAGACAGTCCCACCCTCAC
MT-ND2(R)	human	GAACGTGCTTTGCCCTAGTT
Gnai1(F)	mouse	GCAAGATGATCGACCGCAAC
Gnai1(R)	mouse	CAAGCACGAAAAGTTGGCGA
Gnai2(F)	mouse	TGACTTGGTGCTGGCTGAGGAT
Gnai2(R)	mouse	GATGGAGGTGTCTGTGAACCAC
Gnai3(F)	mouse	GACTACAGGCATTGTGGAGACC
Gnai3(R)	mouse	GGTCGTAATCACTGAGAGCCAC
Arrb1(F)	mouse	CTTCTGTGCTGAGAACCTGGAG
Arrb1(R)	mouse	GGAACTGTCTGGTAGTCTCAGC
Arrb2(F)	mouse	ACGCCGACATTTGCCTCTTCAG
Arrb2(R)	mouse	CACGCTTCTCTCGGTTGTCACT
ARRB1(F)	human	TGGAGAACCCATCAGCGTCAAC
ARRB1(R)	human	AGGCAGATGTCTGCATACTGGC
ARRB2(F)	human	CTGACTACCTGAAGGACCGCAA
ARRB2(R)	human	GTGGCGATGAACAGGTCTTTGC
Pik3r1(F)	mouse	CAAACCACCCAAGCCCACTACT
Pik3r1(R)	mouse	CCATCAGCAGTGTCTCGGAGTT
Pik3r2(F)	mouse	CAGTACAACGCCAAGCTGGACA
Pik3r2(R)	mouse	TGCTGGTGGTAGACCTTGAGCT
Pik3r3(F)	mouse	ACCACGAGTCTCTCGCTCAGTA
Pik3r3(R)	mouse	CCTGATACTGAGAGTGGAACTCC

Pik3ca (F)	mouse	CACCTGAACAGACAAGTAGAGGC
Pik3ca (R)	mouse	GCAAAGCATCCATGAAGTCTGGC
Pik3cb (F)	mouse	CAGTTTGGTGTCATCCTGGAAGC
Pik3cb (R)	mouse	TCTGCTCAGCTTCACCGCATTC
Pik3cd (F)	mouse	ACCATCAGTGGCTCTGCGGTTT
Pik3cd (R)	mouse	GTGGTCTTCTGGGAACTCACCT
PIK3R1 (F)	human	CGCCTCTTCTTATCAAGCTCGTG
PIK3R1 (R)	human	GAAGCTGTCGTAATTCTGCCAGG
PIK3R2 (F)	human	ATGGCACCTTCCTAGTCCGAGA
PIK3R2 (R)	human	CTCTGAGAAGCCATAGTGCCCA
PIK3CA (F)	human	CGCCTCTTCTTATCAAGCTCGTG
PIK3CA (R)	human	GAGCATCCATGAAATCTGGTCGC
Gapdh (F)	mouse	CATCACTGCCACCCAGAAGACTG
Gapdh (R)	mouse	ATGCCAGTGAGCTTCCCGTTCAG
GAPDH (F)	human	GTCTCCTCTGACTTCAACAGCG
GAPDH (R)	human	ACCACCCTGTTGCTGTAGCCAA

shRNA primers

Name	Site	Sequence 5' to 3'
CCL5	1	GGGAGTACATCAACTCTT
CCL5	2	CGCTGTCATCCTCATTGCT
Ccl5	1	CAAGAAATCAGCATTTCAT
Ccl5	2	GTGCCCACGTCAAGGAGTA
CCR1	1	CCCTACAATTTGACTATACTT
CCR3	1	GCTCCGAATTATGACCAACAT
CCR4	1	CCCTTAGGGATCATGCTGTTT
CCR5	1	GCTTCTTAAATGAGAAGGAAT
CCR5	2	CCAGACATTAAAGATAGTCAT
Ccr1	1	GTTTCAAGCTCTAAAGCTA
Ccr3	1	GCTAATATCTACCTGTTCA
Ccr4	1	TGGACTAGGTCTGTGCAAG
Ccr5	1	CCATTTCAGAGGTTTAGGA
Ccr5	2	CCGTTAACTTTCTTCCTTA
PIK3R1	1	GCGCTATGCAATTCTTAATTT
PIK3R2	1	CGCGAGTATGACCAGCTTTAT
PIK3CA	1	GGACCTCAATTCACCTCATAG
ARRB1	1	AGATCTCAGTGCGCCAGTATG
ARRB1	2	GGTCCCAGGTGGTTAACAAAG

siRNA

Name	Site	Target sequance 5' to 3'
Gnai1	1	CAGTACAAGGCAGTGGTCTACAGCA
Gnai2	1	CGCGGGAGGTGAAGTTGCTTCTGTT
Gnai3	1	CCAAAGAAGTGAAGCTGCTGCTGCT
Arrb2	1	CGCGACTTTGTAGATCACCTGGACA
Arrb1	1	GATCTATTACCACGGAGAA
Arrb1	2	GCATCATCGTTTCCTACAA
Pik3r1	1	CCTGGACTTAGAGTGTGCCAAGACA
Pik3r2	1	TGAGCAAGCAGAGCTGGACAGTGAA
Pik3r3	1	GAAATCACGTCTGGGTGAGATTCAT
Pik3cd	1	GGGACCGCGTGAAGAAGCTCATTAA
Pik3ca	1	CCCACGAATCCTAGTGGAATGTTTA
Pik3cb	1	CCACCGGGATTTATATCCAGTTGGA
